# Probabilistic Prognostics and Health Management of Power Transformers Using Dissolved Gas Analysis Sensor Data and Duval’s Polygons

**DOI:** 10.3390/s25216520

**Published:** 2025-10-23

**Authors:** Fabio Norikazu Kashiwagi, Miguel Angelo de Carvalho Michalski, Gilberto Francisco Martha de Souza, Halley José Braga da Silva, Hyghor Miranda Côrtes

**Affiliations:** 1 Department of Mechatronics and Mechanical Systems Engineering, Polytechnic School, University of São Paulo, Avenida Professor Mello de Moraes 2231, São Paulo 05508-030, SP, Brazil; fabionkashiwagi@usp.br (F.N.K.); gfmsouza@usp.br (G.F.M.d.S.); halley.braga@gmail.com (H.J.B.d.S.); hyghor.cortes@edp.com (H.M.C.); 2 EDP Brasil, Avenida Cassiano Ricardo, 1973—Jardim Alvorada, São José dos Campos 12240-540, SP, Brazil

**Keywords:** DGA, forecasting, prognostics and health management, time-series forecasting, uncertainty quantification, power grid reliability

## Abstract

**Highlights:**

**What are the main findings?**

- A probabilistic framework is proposed for transformer fault detection, diagnosis, and prognosis using Dissolved Gas Analysis (DGA) sensor data.
- The method integrates self-adaptive ARIMA forecasting with probabilistic extensions of Duval’s polygons, enabling uncertainty-aware fault classification and failure risk estimation.

**What is the implication of the main findings?**

- The framework improves reliability of transformer condition monitoring by providing early warnings and robust fault evolution tracking.
- It supports risk-based maintenance decisions in smart grid environments, enhancing operational safety and asset lifetime.

**Abstract:**

Power transformers are critical assets in modern power grids, where failures can lead to significant operational disruptions and financial losses. Dissolved Gas Analysis (DGA) is a key sensor-based technique widely used for condition monitoring, but traditional diagnostic approaches rely on deterministic thresholds that overlook uncertainty in degradation dynamics. This paper proposes a probabilistic framework for Prognostics and Health Management (PHM) of power transformers, integrating self-adaptive Auto Regressive Integrated Moving Average modeling with a probabilistic reformulation of Duval’s graphical methods. The framework enables automated estimation of fault types and failure likelihood directly from DGA sensor data, without requiring labeled datasets or expert-defined rules. Dissolved gas dynamics are forecasted using time-series models with residual-based uncertainty quantification, allowing probabilistic fault inference from predicted gas trends without assuming deterministic persistence of a specific fault type. A sequential pipeline is developed for real-time fault tracking and reliability assessment, aligned with IEC, IEEE, and CIGRE standards. Two case studies validate the method: one involving gas loss in an experimental setup and another examining thermal degradation in a 345 kV transformer. Results show that the framework improves diagnostic reliability, supports early fault detection, and enhances predictive maintenance strategies. By combining probabilistic modeling, time-series forecasting, and sensor-based diagnostic inference, this work contributes a practical and interpretable PHM solution for sensor-enabled monitoring environments in modern power grids.

## 1. Introduction

Power transformers are critical assets in electrical substations and modern power grids, where failures can lead to significant operational disruptions, financial losses, and safety risks [1,2]. Their operational lifespan is primarily determined by the condition of the insulation system, which gradually degrades due to electrical, environmental, and mechanical stresses [3].

Beyond operational disruptions, transformer failures can also result in substantial financial losses. For example, distribution-level studies have estimated aggregate impacts of several hundred million euros annually [4], while analyses at the transmission level show that failures of high-voltage units are associated with the highest interruption costs for end-users [5]. From a life-cycle perspective, failures also entail both direct recovery expenses and indirect social and economic losses [6], and reviews consistently highlight the significant financial risks and prolonged outages linked to such events [7]. These figures reinforce the practical relevance of accurate diagnostics and prognostics for such critical assets.

Dissolved Gas Analysis (DGA) is a sensor-based diagnostic technique widely adopted for monitoring oil-immersed transformers, enabling early fault detection through the evaluation of gas concentrations and their evolution over time [8,9,10]. Standard diagnostic models, such as Doernenburg’s method, Rogers’ ratios, and the IEC 60599 and IEEE C57.104 guides, use deterministic gas thresholds to classify fault types [11,12]. Structured graphical approaches like Duval’s Triangle [13,14,15] and Pentagon [16,17,18] offer domain-informed decision support, but still rely on fixed concentration boundaries. This rigidity limits their capacity to capture fault progression dynamics and to provide probabilistic insight into failure risk.

With the advent of online monitoring systems and embedded sensors [19,20], continuous measurement of gas concentrations has become feasible, making real-time prognostics increasingly viable. However, conventional DGA-based classification methods remain categorical and threshold-driven, offering limited support for uncertainty quantification, an essential requirement in modern Prognostics and Health Management (PHM) strategies [21,22].

Artificial intelligence (AI) techniques such as Artificial Neural Networks (ANNs) [23,24,25], fuzzy logic [26,27], Support Vector Machines (SVMs) [28,29,30], and hybrid models [31,32,33] have been proposed to enhance diagnostic performance. While often effective in static classification, these models generally require large labeled datasets, expert-defined rules, or high computational resources. As such, they are not always suitable for continuous or adaptive PHM in industrial settings.

This work introduces a probabilistic framework for transformer health assessment, integrating fault detection, diagnosis, and failure prognosis under uncertainty. The framework combines self-adaptive Auto Regressive Integrated Moving Average (ARIMA) forecasting with a reformulated, uncertainty-aware version of Duval’s diagnostic polygons. By eliminating the need for labeled training data or heuristic rules, it extends existing DGA interpretation methods while remaining compatible with international standards. Gas concentration trends are modeled using dynamically selected ARIMA processes via the Hyndman–Khandakar algorithm [34,35], which enables the probabilistic estimation of fault states and future degradation.

Although ARIMA and its variants have been previously applied to forecast dissolved gas concentrations in transformers, such as SARIMA-based trend prediction considering temperature effects [36], wavelet-ARIMA with non-parametric kernel density estimation for interval forecasts [37], and ensemble learning optimized by Dempster–Shafer theory in comparison with SARIMA [38], these works remain focused on concentration forecasting, and their integration into a structured diagnostic framework has scarcely been addressed. The key innovation of the present study lies in embedding adaptive ARIMA forecasting into Duval’s diagnostic polygons, reformulated as probabilistic models. This enables dynamic fault classification with quantified uncertainty and supports risk-informed prognosis by estimating the probability of failure within a defined time horizon, contributing to reliability-centered maintenance planning.

While some recent studies explore probabilistic models for transformer diagnostics using DGA [39,40], they do not combine time-series forecasting with structured inference, nor do they offer a unified PHM-oriented methodology grounded in industrial practice. By integrating probabilistic forecasting with uncertainty-aware diagnostic inference, the proposed framework supports robust, data-driven decision-making while maintaining computational efficiency. Its continuous adaptability to new operating conditions makes it well-suited for real-time monitoring, offering a practical and scalable solution for transformer health management in operational environments.

The main contributions of this work are as follows:

- A unified probabilistic reformulation of Duval’s diagnostic structures;
- Integration of adaptive time-series forecasting and residual-based uncertainty modeling;
- A sequential and interpretable pipeline encompassing detection, diagnosis, and risk-informed prognosis;
- Compatibility with international reliability standards (IEC, IEEE, CIGRE), ensuring industrial applicability.

The proposed method is validated using published real-world transformer fault data, demonstrating its suitability for operational PHM environments. The remainder of this paper is organized as follows: Section 2 reviews DGA fundamentals, Duval’s methods, and ARIMA modeling. Section 3 details the proposed probabilistic framework. Section 4 presents validation results. Section 5 concludes with key findings and future directions.

## 2. Theoretical Background

This section outlines the core concepts of the proposed framework, including DGA for transformer monitoring, conventional classification methods (e.g., gas ratios and Duval’s polygons), and the fundamentals of self-adaptive ARIMA for gas trend forecasting.

### 2.1. DGA in Transformer Monitoring

The reliability and lifespan of power transformers depend on the condition of their cellulose-based insulation immersed in mineral oil. With proper maintenance, these assets can operate for 25 to 50 years [22]. However, internal stresses (e.g., insulation degradation, partial discharges, localized overheating) and external factors (e.g., lightning, switching surges, overloads) accelerate aging and increase failure risks [22,41].

DGA enables early fault detection by analyzing gas concentrations dissolved in insulating oil. It supports preventive maintenance by identifying gas evolution patterns indicative of insulation aging and fault severity [40]. Recent advances in sensor technology allow continuous online monitoring, improving responsiveness and diagnostic accuracy, while traditional practices rely on periodic oil sampling and laboratory analysis [20].

DGA-based diagnostic frameworks, such as IEC 60599 [11] and IEEE C57.104 [12], classify transformer faults based on both gas composition and concentration levels. They provide structured methodologies to associate gas patterns with specific fault types, such as thermal degradation, electrical discharges, and chemical anomalies. 

According to IEC 60599 [11], thermal faults result from localized or prolonged overheating, whereas electrical faults involve ionization of the insulating medium under high-voltage stress [42,43]. Thermal faults (T1–T3) escalate in severity, from minor aging effects (<300 °C) to severe carbonization and insulation damage (>700 °C). Electrical discharges are similarly classified: low-energy (D1) events cause limited insulation breakdown, whereas high-energy (D2) discharges lead to extensive carbonization and metal erosion, posing immediate risks [11]. The IEEE C57.104 standard [12] complements this framework by incorporating gas trend analysis to monitor fault evolution and proximity to critical thresholds.

Table 1 summarizes the fault types according to gas behavior and temperature thresholds, consistent with the categories currently adopted in international standards and industry practice.

Recent studies [17,44,45] have introduced additional fault subtypes, particularly new sub-zones in Duval’s Triangles and Pentagons to differentiate arcing in paper versus oil and to identify carbonization of paper insulation. These valuable refinements, however, are not yet consolidated in IEC 60599 (2022) [11] or IEEE C57.104 (2019) [12], and remain outside current industry standards.

DGA interpretation is guided by standardized methodologies that associate gas thresholds and temporal patterns with fault types. While IEEE and IEC rely on absolute concentration limits and trend analysis, CIGRE enhances predictive capability by incorporating historical failure data and probabilistic thresholds [11,12,46]. Despite sharing core principles, IEEE, IEC, and the CIGRE Joint Task Force D1.01/A2.11 differ in key aspects:

- Concentration Thresholds: IEEE and IEC use decision matrices; CIGRE integrates historical data to estimate failure probabilities.
- Rate-of-change Analysis: IEEE emphasizes baseline trends; IEC focuses on distinguishing aging from abnormal degradation; CIGRE introduces explicit sampling intervals and slope thresholds.
- Pre-Failure Limits: Only CIGRE defines pre-failure gas concentration thresholds, allowing RUL estimation. IEEE and IEC recommend increased monitoring when elevated gas levels are detected [11,12,15,46].

Among the three, CIGRE uniquely offers a structured methodology for pre-failure risk assessment, supporting long-term asset management and enabling utilities to intervene before transformers reach critical conditions [46].

### 2.2. DGA Fault Classification Methods

The classification of transformer faults using DGA has been a central topic in diagnostic research for decades, leading to the development of ratio-based and graphical methods. These techniques analyze the composition and proportions of dissolved gases to detect failures early and support maintenance planning strategies [22].

Gas ratio methods are among the earliest and most established approaches. They rely on predefined relationships between gases such as hydrogen (H_2_), methane (CH_4_), ethane (C_2_H_6_), ethylene (C_2_H_4_), and acetylene (C_2_H_2_) to classify faults, including thermal overheating and electrical discharges [11,42]. Despite their simplicity and field applicability, methods like the Doernenburg Ratio Method (DRM), Rogers Ratio Method (RRM), and IEC Ratio Method (IRM) often yield inconclusive results in mixed or evolving fault conditions due to rigid thresholds and limited sensitivity [43].

To overcome these limitations, graphical techniques were developed to map gas concentrations into predefined fault zones. The most widely adopted is the Duval Triangle Method (DTM 1), which uses C_2_H_2_, C_2_H_4_, and CH_4_ to distinguish between thermal and electrical faults [13]. However, to improve fault resolution, additional models were introduced. Duval Triangle 4 (DTM 4) includes H_2_, CH_4_, and C_2_H_6_ for better detection of low-energy faults, while Triangle 5 (DTM 5) focuses on high-temperature events using C_2_H_2_, C_2_H_4_, and C_2_H_6_ [12].

Duval Pentagon models further expand diagnostic resolution. Pentagon 1 (DPM 1) incorporates all five gases to improve differentiation of low-energy discharges and mild overheating [44]. Pentagon 2 (DPM 2) enables more granular classification of thermal faults (T3-H, C, O, S) and discharge types (PD, D1, D2), making it particularly valuable for aging assessment [16,47].

Comparative studies confirm the superiority of Duval’s graphical methods over ratio-based ones. Liu et al. (2015) [48] reported 93.94% accuracy for DTM 1, while Faiz & Soleimani (2017) [49] showed over 90% performance in arcing fault classification using Duval Triangles. Despite its robustness, DTM 1 lacks sensitivity to certain gases like C_2_H_6_ and H_2_, a limitation addressed by the extended models [50].

Overall, the Duval Triangle and Pentagon series remain the most interpretable and accurate tools for traditional DGA. Their structured design, validation history, and adaptability to refinements make them ideal candidates for integration into hybrid or probabilistic diagnostic frameworks [10,22].

In parallel, recent advances in machine learning and AI have improved DGA-based classification, especially in borderline or ambiguous cases. These data-driven models learn from historical patterns and real-time monitoring, reducing reliance on static thresholds and enhancing adaptability [20,51]. Neural networks have demonstrated superior performance in identifying nonlinear gas-fault relationships, outperforming conventional methods in fault differentiation [8,23,24,25,52,53,54].

Despite their high accuracy, purely AI-based models often lack interpretability, require extensive labeled datasets, and involve significant computational overhead, limiting their applicability in continuous monitoring of critical infrastructure. These constraints are particularly relevant in industrial contexts where transparency, standard compliance, and scalability are essential.

Consequently, there is growing interest in hybrid approaches that balance data-driven adaptability with structured diagnostic reasoning grounded in expert knowledge. These approaches, such as AI-Duval models, integrate graphical tools with adaptive algorithms to dynamically refine decision boundaries while preserving interpretability [39,40,55].

Following this direction, this study introduces a framework that integrates probabilistic modeling, uncertainty quantification, and adaptive machine learning to combine the strengths of statistical learning and structured expert-based inference. The result is a scalable, interpretable, and risk-informed solution for transformer health assessment.

### 2.3. Self-Adaptive ARIMA Modeling

Accurate prediction of gas concentration trends in DGA is essential for assessing the severity and progression of power transformer failures. In this sense, ARIMA models offer a robust statistical framework for time series modeling and can dynamically adapt to changes in gas behavior after failure initiation [36,37,38,56,57].

ARIMA extends the Autoregressive Moving Average (ARMA) model by incorporating differencing to handle non-stationary data. Unlike Markovian stochastic models, where future states depend solely on the current state, ARIMA captures dependencies across both past and present observations [58]. This ability to model autocorrelation differentiates ARIMA from simpler methods like Exponential Smoothing, which may fail to capture long-term sequential dependencies.

An ARIMA model is defined by three parameters: (*p*, *d*, *q*), where *p* represents the number of autoregressive terms (dependence on past observations), *d* is the degree of differencing required for stationarity, and *q* is the order of the moving average component (dependence on past forecast errors) [59]. If seasonal components are considered, the model extends to ARIMA (*p*, *d*, *q*)(*P*, *D*, *Q*)*_m_*, where (*P*, *D*, *Q*) defines seasonal parameters and *m* denotes periodicity [34].

Each component of the ARIMA model serves a specific function: the autoregressive (AR) term models linear dependencies on past values, the integrated (I) component ensures stationarity through differencing, and the moving average (MA) term captures dependencies on past forecast errors, improving adaptability. These elements form a flexible framework, where any unexplained variance is treated as additive noise.

A generalized ARMA model can be expressed as presented by Equation (1),

(1) $$y_{t}-\sum_{i=1}^{p}\varphi_{i}\;y_{t-i}=\varepsilon_{t}+\sum_{j=1}^{q}\theta_{j}\;\varepsilon_{t-j}$$

where *y_t_* is the time series value at time *t*, *j_i_* are the autoregressive coefficients, *q_j_* are the moving average coefficients, and *e_t_* is white noise. For ARIMA models, differencing is applied using the lag operator *L*(·), extending Equation (1) to Equation (2), where *j*(*L*) is the autoregressive component, given by Equation (3), and *q*(*L*) is the moving average component, given by Equation (4) [60].

(2) $$\varphi \left(L\right){\left(1-L\right)}^{d}y_{t}=\theta \left(L\right)\varepsilon_{t}$$

(3) $$\varphi \left(L\right)=1-\sum_{i=1}^{p}\varphi_{i}\;L^{i}$$

(4) $$\theta \left(L\right)=1+\sum_{j=1}^{q}\theta_{j}\;L^{j}$$

To improve stationarity and adherence to normality and homoscedasticity assumptions, the Box–Cox transformation is applied, as shown in Equation (5):

(5) $$y_{t}^{\left(\lambda \right)}=\left\{\begin{array}{c} \frac{y_{t}^{\lambda }-1}{\lambda }\;\;\;,\;\;\;\lambda \neq 0\\ \ln\left(y_{t}\right)\;\;\;,\;\;\;\lambda =0 \end{array}\right.$$

where *λ* is optimized to maximize log-likelihood. Adjustments are made when zero or negative values occur [61], for instance, by adopting the shifted version of the Box–Cox transformation [62]. In DGA forecasting, this transformation is beneficial for stabilizing variance, especially when gas levels exhibit exponential growth or abrupt shifts following fault detection, thereby enhancing the performance of the ARIMA model.

**Figure 1:**
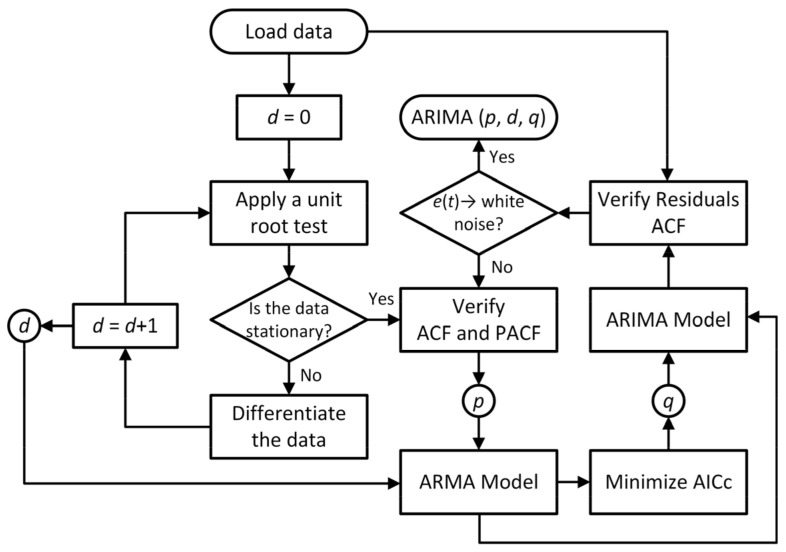
ARIMA (*p*, *d*, *q*) model selection algorithm.

Traditional ARIMA models require manual parameter selection, but self-adaptive ARIMA employs automated hyperparameter optimization, enabling dynamic updates as new data arrive. This adaptability improves real-time forecasting and enhances fault prognosis accuracy. In this framework, the Hyndman-Khandakar algorithm [35] automates model selection through an iterative process (Figure 1). It begins by testing stationarity via unit root tests and applying differencing as needed to determine *d*. The Autocorrelation Function (ACF) and Partial Autocorrelation Function (PACF) assist in selecting *p* and *q*, followed by model evaluation using the corrected Akaike Information Criterion (AICc) [63]. Residual diagnostics are then used to verify that white noise assumptions are met, with parameter refinement applied if autocorrelation persists [58].

Once optimized, the ARIMA model generates forecasts for a defined horizon *h* beyond the current time *t*, following Equation (6):

(6) $${\left(1-L\right)}^{d}y_{\tau +h|\tau }=\left\{\begin{array}{c} \sum_{i=1}^{p}\varphi_{i}\;L^{i}{\left(1-L\right)}^{d}y_{\tau +h|\tau }+\sum_{j=1}^{q}\theta_{j}\;L^{j}\varepsilon_{\tau +h|\tau }\;\;\;;\;\;\;h\leq q\\ \sum_{i=1}^{p}\varphi_{i}\;L^{i}{\left(1-L\right)}^{d}y_{\tau +h|\tau }\;\;\;;\;\;\;h>q \end{array}\right.$$

where future residuals *e_t_*_+*h|t*_ are unknown, impacting long-term accuracy when *h* < *q*. Prediction intervals (e.g., 95% confidence) account for uncertainty, increasing as *h* extends. If residuals deviate from normality, bootstrap resampling is applied to generate robust intervals under constant variance and uncorrelated assumptions [64,65].

Despite certain limitations, such as sensitivity to stationarity, reliance on linear assumptions, and reduced performance under heteroscedastic or nonlinear conditions [66], ARIMA remains an efficient and interpretable choice for forecasting, especially considering short-term horizons. Due to this efficiency, hybrid models combining ARIMA with nonlinear approaches have been proposed to enhance adaptability while preserving interpretability. Compared to more sophisticated techniques, such as Long Short-Term Memory (LSTM) networks that capture complex temporal dependencies, ARIMA is lightweight and better suited for real-time monitoring in data-limited industrial environments [37,67].

#### Comparing ARIMA with Alternative Forecasting Methods for DGA

Accurate DGA forecasting is essential for tracking gas accumulation and assessing fault progression. Forecasting models must balance process constraints, reliability, and interpretability. Key requirements include:

- Autocorrelation: Gas behavior follows degradation patterns rather than random fluctuations. Models must capture autocorrelation caused by thermal decomposition and electrical discharges, as well as structured decreases after remediation [68].
- Interpretability: As DGA supports diagnosis and maintenance planning, models must provide clear insights into gas trends. Black-box approaches limit transparency and hinder operational adoption.
- Computational Efficiency: Real-time monitoring demands models with acceptable accuracy and low computational cost. Lightweight statistical methods are often preferable to data-hungry deep learning techniques.

Different methods address these trade-offs with varying success. Holt-Winters exponential smoothing is efficient but fails to model autocorrelation, limiting its utility for DGA [34]. Bayesian Structural Time Series (BSTS) captures dependencies, but adds complexity and interpretability issues [69]. Gaussian Process Regression (GPR) supports probabilistic forecasts but demands large datasets rarely available in practice [70].

Deep learning models, such as LSTMs, GRUs, and Transformers, model complex temporal dependencies but require substantial data and high computational power, making them less suitable for real-time, data-limited environments [71]. While LSTMs often outperform ARIMA in long-term forecasts, ARIMA offers a balanced solution for short-term horizons due to its efficiency and transparency [71]. Recent studies confirm ARIMA’s strong performance in time series forecasting under constraints of limited data and high interpretability [37,38].

Table 2 summarizes these methods based on their ability to model autocorrelation, handle small datasets, provide interpretability, and support real-time applications, highlighting trade-offs between model complexity and operational feasibility. Given these considerations, Self-Adaptive ARIMA offers a well-balanced solution, combining adequate accuracy, transparent interpretation, and low computational cost, making it particularly suitable for real-time DGA forecasting in industrial settings where adaptability and clarity are essential

## 3. Proposed Framework

**Figure 2:**
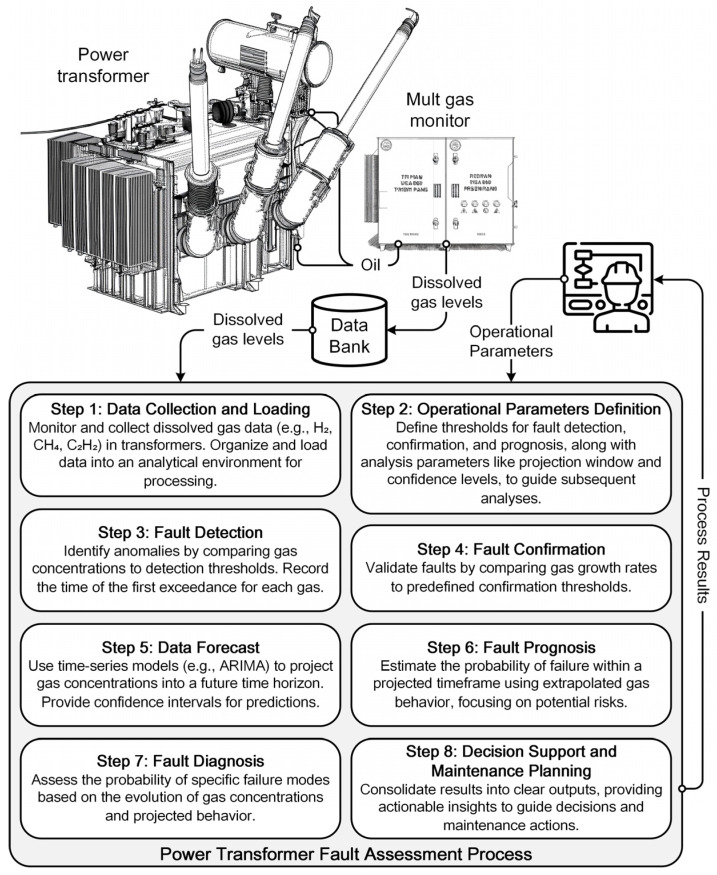
Illustrative Diagram of the Proposed Framework.

The proposed framework comprises eight stages, integrating time-series forecasting and probabilistic fault inference for transformer condition assessment. It supports risk-informed decisions by processing DGA data from acquisition to maintenance planning. Figure 2 provides an overview of the full pipeline.

### 3.1. Data Collection and Loading

The monitoring process involves the periodic acquisition of dissolved gas concentrations (H_2_, CH_4_, C_2_H_2_, C_2_H_4_, and C_2_H_6_) through continuous online sensors. Each data point is time-stamped and stored in a structured database, ensuring traceability for further analysis. Before modeling, the dataset undergoes preprocessing to remove missing values and detect inconsistencies, preserving statistical robustness. To optimize computational efficiency, only the five primary gases and their timestamps are retained.

### 3.2. Operational Parameter Definition

The framework defines key operational parameters for fault detection, confirmation, and prognosis, ensuring a structured and standardized analysis. These thresholds classify transformers as operating normally, developing faults, or at high failure risk.

Detection thresholds, indicating initial gas concentration anomalies, are derived from IEEE C57.104 [12], IEC 60599 [11], or CIGRE Joint Task Force D1.01/A2.11 [46]. Typically associated with Typical Gas Concentration (TGC) values, these thresholds represent the maximum acceptable gas levels, exceeded by only 10% of transformers, prioritizing maintenance for the most at-risk units [11].

Confirmation thresholds, which determine whether an anomaly evolves into a fault, are based on Typical Rates of Gas Increase (TRGI), ensuring consistency across regulatory standards. For failure prognosis, the Pre-Failure Gas Concentration (PFGC), defined by CIGRE [46], identifies transformers at high risk over future time horizons. While CIGRE also introduces Alarm Gas Concentration (AGC) values, these are not considered in this framework. A comprehensive reference for threshold values and degradation limits is provided by Duval (2008) [15], facilitating comparative analysis and improving risk assessments.

Beyond these thresholds, additional parameters must be defined to support the forecasting and diagnostic procedures. The analysis window (*w*), which determines the number of past observations used for time series modeling, should be selected to ensure statistical robustness while minimizing the influence of short-term fluctuations. Its length may vary depending on data resolution and fault progression dynamics. In practice, windows of around 50 observations or more provide a useful estimate of the autocorrelation function, which is a fundamental step in ARIMA model identification [72]. The projection window (*h*) defines the forecasting horizon for failure prognosis and predictive maintenance planning. Confidence levels, typically set at 95%, establish probabilistic bounds, supporting a balanced assessment of false positives and false negatives in transformer fault evaluation.

### 3.3. Fault Detection

Anomalies are detected when gas concentrations exceed predefined detection thresholds. Upon the first occurrence of an abnormal reading, the system records the detection time (*t*_detection_) and triggers an alert for further verification.

### 3.4. Fault Confirmation

Fault confirmation is conducted by analyzing gas growth rates over time. A fault is confirmed when the growth rate exceeds the confirmation threshold, defined by the TRGI for each gas. Since TRGI values are specified on an annual basis, whereas data collection typically occurs hourly, appropriate scaling is necessary.

Dividing the annual TRGI by 8766 (the number of hours in a year) provides an hourly equivalent, but applying this to single-step comparisons yields thresholds that are too narrow, resulting in unreliable assessments. To address this, fault confirmation leverages the same analysis window (*w*) defined in Section 3.2, aggregating multiple observations and computing a more stable estimate of gas growth.

Upon detecting an anomaly, the system retrospectively evaluates the last (*w*) observations before *t*_detection_. If at least one gas exhibits a growth rate exceeding its adjusted TRGI value, the fault is confirmed, and the confirmation time is recorded as *t*_confirmation_. If no fault is detected, the transformer enters enhanced monitoring mode, where each new observation triggers a re-evaluation until confirmation occurs.

Once a fault is confirmed, the reference time (*τ*) for gas concentration forecasting is established, as defined in Equation (7), and the monitored gas concentration data are extrapolated for further analysis. This definition ensures that forecasting begins only after both fault detection and confirmation have occurred, thereby preventing premature extrapolation from unverified signals and providing a reliable basis for prognostic analysis.

(7) $$\tau =\max\left(t_{\mathrm{detection}},t_{\mathrm{confirmation}}\right)$$

### 3.5. Data Forecast

Once a fault is confirmed, ARIMA models are employed to forecast gas concentration trends. The process begins by selecting a historical data window (*w*) extending backward from the reference fault time *τ*.

A Box–Cox transformation is applied when necessary to stabilize variance and improve normality, optimizing *λ* to maximize likelihood. The Hyndman-Khandakar algorithm then automates ARIMA model selection, incorporating unit root tests, differencing operations, and AICc-based model evaluation. A stepwise search method [73,74] ensures computational efficiency while exploring optimal parameter configurations.

Once the model is defined, it is trained using data up to *t*, followed by residual diagnostics to confirm adherence to white noise properties and absence of autocorrelation. The Shapiro–Wilk test [75] evaluates normality, ensuring the residuals fit an expected distribution. If residuals deviate from normality, corrective measures such as Box–Cox transformations are applied to improve forecast suitability. The transformed residuals are then used to define kernel density distributions for probabilistic forecasting of gas concentrations.

Additional diagnostic tests ensure robustness:

- The Ljung–Box test [76,77] verifies residual independence, confirming that the ARIMA model effectively captures temporal dependencies. The number of lags is set to $\sqrt{w}$ to maintain diagnostic robustness without overfitting.
- The Lagrange Multiplier (LM) test for ARCH [78] assesses variance stability, identifying potential heteroskedasticity or volatility clustering. The number of lags is also set to $\sqrt{w}$, consistent with the Ljung–Box test.

Once validated, the ARIMA model forecasts gas concentration values over the defined horizon *h*, ensuring accuracy in industrial applications. The forecasting process adapts to different time horizons:

- For short-term horizons (*h* ≤ *q*), predictions incorporate past residuals and autoregressive terms, capturing short-term deviations.
- For longer horizons (*h* > *q*), the model relies exclusively on autoregressive components, iteratively using prior forecasts as inputs to generate subsequent values.

For non-stationary series (*d* < 0), inverse differencing restores forecasts to the original scale, preserving consistency with historical data. Confidence intervals (typically 95%) quantify uncertainty, with prediction ranges widening over extended horizons. These intervals support maintenance planning, providing probabilistic bounds within which actual concentrations are expected to fall.

If a Box–Cox transformation was applied during training, forecasts are adjusted back to the original scale using Equation (8).

(8) y^t=λ⋅y^tλ+11λ   ,   λ≠0expy^tλ   ,   λ=0

where ${\hat{y}}_{t}^{\left(\lambda \right)}$ is the forecasted value in the transformed space. However, since this naïve retransformation can lead to systematic underestimation in the presence of residual variance, bias-corrected versions are recommended.

Specifically, for *λ* = 0 (log transformation), bias correction via residual variance prevents systematic underestimation (Equation (9)).

(9) $${\hat{y}}_{t}=exp\left[log\left({\hat{y}}_{t}^{\left(\lambda \right)}+1\right)+\frac{\sigma_{\varepsilon }^{2}}{2}\right]$$

where $\sigma_{\varepsilon }^{2}$ is the residual variance.

For *λ* ≠ 0, a Taylor expansion adjustment (Equation (10)) accounts for nonlinearity.

(10) y^t=λ⋅y^tλ+11λ⋅expλ2σε22

These corrections ensure that forecasts accurately represent the mean in the original space, particularly in high-variance scenarios, mitigating systematic biases. In practice, Equation (8) is rarely used alone; bias-corrected forms (Equations (9) and (10)) are recommended whenever residual variance is non-negligible.

Following the ARIMA-based forecast, probabilistic distributions of gas concentrations are constructed for each time step in *h*, aligning residual kernels with ARIMA projections. If normality is not confirmed, the Box–Cox transformation is reapplied, using the same optimized *λ* parameter from the ARIMA model selection. While this transformation does not guarantee normality, it ensures that the residuals are statistically suitable for kernel density estimation, aligning them with the ARIMA forecasts. If normality is confirmed, the residuals are used directly.

To align the residual kernel with ARIMA projections, key statistical parameters—including the mean and quantiles (e.g., 2.5% and 97.5%) of the ARIMA forecasts—are computed at each time step within *h*, defining the lower and upper bounds of the confidence interval. The residual distributions are then scaled and translated to match these characteristics, ensuring they accurately capture both uncertainty in residuals and the forecast dynamics of gas concentrations over time.

For a given Confidence Level (CL), the Lower Bound (*LB*) and Upper Bound (*UB*) of the forecast interval over *h* steps are computed using Equations (11) and (12). When CL = 95%, these correspond to *Q*_2.5%_ and *Q*_97.5%_ of the ARIMA forecast distribution.

(11) $$LB=Q_{\frac{1-\mathrm{CL}}{2}}$$

(12) $$UB=Q_{\frac{1+\mathrm{CL}}{2}}$$

The adjustment process includes two key transformations:

- Scaling to match the spread of the ARIMA projections, where the scale factor (*SF*) at time *t* is given by Equation (13).

(13) $$SF_{t}=\frac{UB_{\mathrm{ARIMA},t}-LB_{\mathrm{ARIMA},t}}{UB_{\mathrm{Residuals}}-LB_{\mathrm{Residuals}}}$$

where *UB*_ARIMA,*t*_ and *LB*_ARIMA,*t*_ represent the upper and lower quantiles of the ARIMA projections at time *t*, and *UB*_Residuals_ and *LB*_Residuals_ are the corresponding quantiles from the residual kernel distribution.

- Translation to align the residual kernel’s mean with the ARIMA forecast mean at each *t*, using the translation factor (TF) from Equation (14).

(14) $$TF_{t}={\mathrm{Mean}}_{\mathrm{ARIMA},t}-\left({\mathrm{Mean}}_{\mathrm{Residuals}}\cdot SF_{t}\right)$$

where Mean_ARIMA,*t*_ is the mean of the ARIMA forecast at time *t*, and Mean_Residuals_ is the mean of the residual kernel.

The final adjusted kernel distribution at *t* is obtained using Equation (15), ensuring that it accurately captures both residual variability and ARIMA forecast trends. These adjusted distributions form the basis for failure probability estimation and probabilistic fault classification using Duval’s methods.

(15) $${\mathrm{Adjusted}\;\mathrm{Kernel}}_{t}=\mathrm{Residual}\;\mathrm{Kernel}\cdot SF_{t}+TF_{t}$$

### 3.6. Fault Prognosis

After forecasting gas concentrations, failure prognosis estimates the probability of each monitored gas exceeding its Pre-Failure Gas Concentration (PFGC) over the forecast horizon *h*. Unlike deterministic approaches based solely on projected mean values, this methodology employs a probabilistic assessment, leveraging the adjusted kernel distributions constructed in the previous step.

At each future time step, failure probability is calculated as the likelihood that the gas concentration surpasses its PFGC threshold. This probability is derived using the Empirical Cumulative Distribution Function (ECDF), evaluated at the PFGC value for each gas, as given by Equation (16):

(16) $$P_{\mathrm{failure}}\approx 1-\hat{F}\left(\mathrm{PFGC}\right)$$

where $\hat{F}\left(\mathrm{PFGC}\right)$ represents the ECDF evaluated at the PFGC value of the respective gas.

For each time step within *h*, this calculation involves:

- Generating the ECDF from the adjusted kernel distribution at time *t*.
- Evaluating the ECDF at the predefined PFGC value.
- Computing the complement of the ECDF to determine the probability of gas concentration exceeding PFGC.

This process generates a probabilistic failure risk profile, enabling a time-based reliability assessment of the transformer. Failure probabilities are stored alongside their corresponding forecasted time points, ensuring robust estimates by aligning the residual kernel with ARIMA projections. By incorporating uncertainty and temporal dependencies, this approach supports data-driven predictive maintenance and failure mitigation strategies.

### 3.7. Fault Diagnosis

Transformer fault diagnosis is performed using a probabilistic extension of Duval’s methods [14,16,17,44], classifying failure modes based on relative gas concentrations. Unlike traditional approaches that rely on fixed thresholds, this framework treats gas concentration ratios as random variables, employing Monte Carlo simulations to estimate probability distributions of fault types over time.

#### 3.7.1. Duval’s Triangles Probabilistic Analysis

Duval’s Triangles classify failure types based on three-gas ratios, with each triangle using different gas combinations, as previously outlined. Given the probabilistic nature of gas forecasts, these ratios are treated as random variables, ensuring that uncertainty in gas concentrations is incorporated into the diagnostic process.

For each time step *t*, the normalized gas proportions *R_i_*_,*t*_ are computed as given in Equation (17):

(17) $$R_{i,t}=\frac{C_{i.t}}{C_{1.t}+C_{2.t}+C_{3.t}}$$

where *C_i_*_,*t*_ represents the concentration of gas *i* at time *t*. These proportions satisfy the constraint in Equation (18), ensuring interdependence among the three gas ratios. Since one proportion directly determines the others, assuming independence would lead to incorrect probability estimates. Therefore, a joint probability approach must be used to accurately compute the likelihood of failure modes.

(18) $$R_{1.t}+R_{2.t}+R_{3.t}=1$$

Each Monte Carlo iteration generates a set of gas proportions, forming probability distributions at each time step. The failure mode probability *P_F,t_* is then computed as the likelihood that a sampled gas proportion satisfies the classification boundaries of a given failure mode. Mathematically, this is determined using the Joint Empirical Cumulative Distribution Function (JECDF), as given in Equation (19):

(19) $$P_{F,t}\approx {\hat{F}}_{R_{1,t},R_{2,t},R_{3,t}}\left(I_{1},I_{2},I_{3}\right)$$

where ${\hat{F}}_{R_{1,t},R_{2,t},R_{3,t}}\left(I_{1},I_{2},I_{3}\right)$ represents the JECDF of the gas proportions, and *I_i_* denotes the classification boundaries for each failure mode within the Duval Triangle model.

A complete formulation of failure mode probabilities for Duval’s Triangles, considering gas-specific classification regions, is provided in Appendix A, ensuring a rigorous probabilistic fault classification approach.

#### 3.7.2. Duval’s Pentagons Probabilistic Analysis

The probabilistic approach used for Duval’s Triangles extends to Duval’s Pentagons, which classify failure modes based on five gas proportions: H_2_, CH_4_, C_2_H_2_, C_2_H_4_, and C_2_H_6_. These pentagon-based models provide greater differentiation for low-energy discharges, high-temperature thermal faults, and carbonization effects, introducing an additional dimension to the diagnostic space.

Unlike the triangle-based analysis, which directly compares gas concentrations to threshold limits, the pentagon method maps gas concentrations onto a two-dimensional coordinate system. This transformation allows failure modes to be evaluated based on geometric regions within the pentagon space.

For each time step *t*, the coordinates (*x_g_*_,*t*_, *y_g_*_,*t*_) for each gas *g* are computed using Equations (20) and (21):

(20) $$x_{g,t}=100\cdot R_{g,t}\cdot \cos\left(\theta_{g}\right)$$

(21) $$y_{g,t}=100\cdot R_{g,t}\cdot \sin\left(\theta_{g}\right)$$

where *R_g,t_* is the normalized gas proportion (Equation (22)), and *θ_g_* is the angular position of gas *g* in the pentagon, defined in Table 3.

(22) $$R_{g,t}=\frac{C_{g,t}}{\sum_{i=1}^{N}C_{i,t}}$$

where *C_g,t_* represents the concentration of gas *g*, and *N* = 5 is the total number of gases considered in the pentagon model.

Once gas proportions are mapped, a Monte Carlo simulation estimates the probabilistic distribution of the centroid position within the pentagon. For each iteration, gas concentrations are sampled using empirical kernel density estimates, and their coordinates are computed using the transformation equations.

The pentagon is divided into four triangular subregions, preserving the gas order defined in Table 3 (H_2_ → C_2_H_6_ → CH_4_ → C_2_H_4_ → C_2_H_2_). The area of each sub-triangle is computed using the determinant method (Equation (23)), and the total pentagon area is calculated in Equation (24):

(23) $$A_{i}=x_{i}\cdot y_{i+1}-x_{i+1}\cdot y_{i}\;\;\;\;,\;\;\;\;\;\mathrm{for}\;\;i=1,2,3,4$$

(24) $$A_{\mathrm{total}}=\frac{1}{2}\sum_{i=1}^{4}A_{i}$$

where the last gas (*i* = 5) wraps around to connect with *i* = 1.

The centroid coordinates (*C_x_*, *C_y_*) are then computed using Equations (25) and (26):

(25) $$C_{x}=\frac{1}{6\cdot A_{\mathrm{total}}}\sum_{i=1}^{4}\left(x_{i}+x_{i+1}\right)A_{i}$$

(26) $$C_{y}=\frac{1}{6\cdot A_{\mathrm{total}}}\sum_{i=1}^{4}\left(y_{i}+y_{i+1}\right)A_{i}$$

This ensures that the centroid’s position accounts for the relative gas proportions in a statistically robust manner.

After computing the probabilistic distribution of (*C_x_*, *C_y_*), fault classification is performed by determining the probability that the centroid falls within predefined fault regions in Duval’s Pentagon. Unlike deterministic classification, this probabilistic approach captures interdependencies between centroid coordinates, which arise due to gas proportion normalization.

To model these correlations accurately, the probability of a failure mode occurring at time step *t* is computed using the JECDF, as given in Equation (27):

(27) $$P_{F,t}\approx {\hat{F}}_{C_{x,t},C_{y,t}}\left(I_{x},I_{y}\right)$$

where ${\hat{F}}_{C_{x,t},C_{y,t}}\left(I_{x},I_{y}\right)$ represents the JECDF of the gas proportions, and *I_x_* and *I_y_* are the classification region boundaries for each Duval Pentagon model.

The complete formulation of failure mode probabilities for Duval’s Pentagons, considering gas-specific classification regions, is provided in Appendix A.

### 3.8. Decision Support and Maintenance Planning

Accurate DGA interpretation is crucial for assessing power transformer health, requiring structured fault classification that accounts for measurement uncertainty and probabilistic centroid estimation. To ensure reliable diagnosis, Duval’s graphical models follow a hierarchical approach, applied in the following sequence:

1. Primary Fault Diagnosis (Duval’s Triangle 1 & Pentagon 1)—Identifies six fundamental failure modes:
  − PD: Partial discharges (corona);
  − D1: Low-energy discharges;
  − D2: High-energy discharges;
  − T1: Low-temperature thermal faults (<300 °C);
  − T2: Intermediate-temperature thermal faults (300–700 °C);
  − T3: High-temperature thermal faults (>700 °C).

If the diagnosis is clear and consistent, further analysis is not required.

2. Refinement for Mild Overheating (Duval’s Triangle 4 & Pentagon 2)—Applied if T1, T2, or PD is detected to differentiate:
  − S: Stray gassing (<200 °C);
  − O: Overheating of paper or oil (<250 °C, without significant degradation);
  − PD: Specific types of partial discharges.

This step helps exclude misleading diagnoses in cases of mild overheating.

3. Refinement for High-Temperature Faults (Duval’s Triangle 5 & Pentagon 2)—Applied if T2 or T3 is detected to distinguish:
  − C: Paper carbonization (>300 °C);
  − T3-H: High-temperature thermal fault affecting only the insulating oil.

This step ensures the correct differentiation between failures involving solid insulation degradation and those occurring exclusively in the insulating fluid.

4. Convergence Analysis & Multiple Fault Detection—After applying graphical models, results are assessed for consistency:
  − Single failure: If all models agree on the same failure mode.
  − Multiple failures: If discrepancies exist between triangles and pentagons, requiring further analysis of centroid distributions and interactions.

Once failure modes are identified, their probabilities are estimated, moving beyond deterministic classification to probabilistic assessment. Based on failure probability, different maintenance strategies are adopted:

- Low probability: Predictive maintenance with continued DGA monitoring.
- Moderate probability: Transformer inspections, visual assessments, and potential offline testing.
- High probability: Corrective actions, including component replacement or scheduled shutdown.

The final decision considers transformer criticality within the power grid and the dissolved gas evolution rate, as outlined in IEC 60599 [11,42]. The combined use of Duval’s Triangles and Pentagons enhances failure prediction accuracy, providing a robust framework for transformer maintenance and operational decision-making.

## 4. Validation and Results

The proposed probabilistic framework is validated through two case studies based on real failure data. Gas concentration values were extracted from graphical representations and discretized into hourly intervals to create structured datasets for modeling and fault assessment. Since the datasets were already preprocessed, the application of the framework begins at the Operational Parameter Definition stage. Additionally, while the analysis in both cases is conducted retrospectively, considering all available observations from the dataset’s start to the fault confirmation point, in industrial settings, the framework would operate sequentially, using only the data available at each time step as new measurements are acquired.

The first case investigates gas losses in an experimental oil tank, showing how diffusion in free-breathing systems can distort DGA-based classification. The second involves a thermal fault in a 345 kV transformer, with paper insulation carbonization confirmed after shutdown.

In the case study, all available observations are analyzed retrospectively, from the dataset’s beginning up to the fault detection and confirmation points. In practice, however, industrial applications would perform confirmation sequentially, using only the data available at each time step as new measurements are acquired.

### 4.1. Case Study 1: Gas Losses in an Experimental Oil Tank Setup

**Figure 3:**
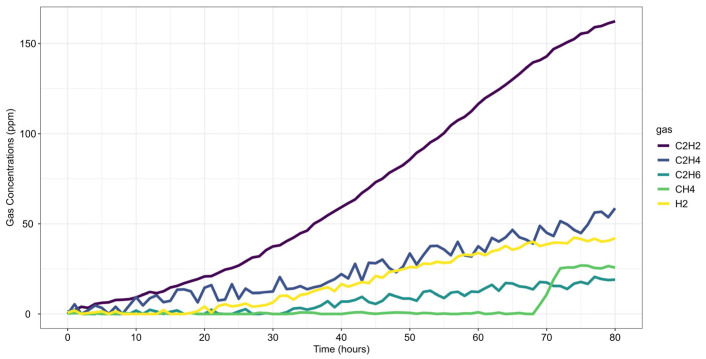
Case Study 1: Gas Concentrations Over Time.

The first case study analyzes gas loss mechanisms in an experimental oil tank setup, as reported by Riedmann (2023) [41]. It examines dissolved gas behavior in free-breathing transformer systems, where gas diffusion affects DGA-based fault classification. The study identifies an electrical arcing fault, characterized by significant C_2_H_2_ generation, highlighting how gas losses impact fault interpretation and condition assessment. Gas concentrations were recorded over the last 80 h of operation before the shutdown, as presented in Figure 3.

The first step in the case analysis involves defining the operational parameters used for detection and confirmation. Thresholds were based on Level 2 limits for gas concentration (ppm) and gas increase rate (ppm/year), representative of an average CIGRE/IEC power transformer. Pre-failure gas concentration limits (ppm) were also defined according to CIGRE standards [15], as summarized in Table 4.

The analysis window (*w*) was set to 48 observations, corresponding to 48 h of historical data. This choice balances statistical robustness and responsiveness: it provides enough data points to allow reliable ARIMA identification, close to the guideline of approximately 50 observations commonly recommended for a useful estimate of the autocorrelation function [72], while remaining short enough to capture relevant variations in gas behavior without introducing excessive lag.

The projection window (*h*) was set to 32 h, constrained by the available data and the timing of fault detection within the dataset.

Regarding fault detection, the first gas to exceed the concentration threshold was C_2_H_2_, at time step 15. For fault confirmation, the first gas to surpass the rate-of-increase threshold was also C_2_H_2_, at time step 48. Thus, the reference time for failure prognosis was established at step 48. As discussed in Section 3.4, fault confirmation relies on the analysis window (*w*) to mitigate distortions introduced when converting annual gas growth rates into hourly equivalents.

Once the fault is confirmed, the framework proceeds to the Data Forecast stage. Due to data limitations, the ARIMA model was trained starting at time step 48, using a single reference point for forecasting. Table 5 presents the selected ARIMA parameters and results of residual diagnostics.

The ARIMA model parameters comprise the order (*p*, *d*, *q*) and the Box–Cox transformation parameter (*λ*), employed to stabilize variance. Model adequacy is assessed by the log-likelihood, where higher values denote superior fit, and by the Root Mean Square Error (RMSE), where lower values indicate greater forecasting accuracy. Residual properties are further examined through statistical tests: the first-order autocorrelation (ACF1), expected to approximate zero for white noise; the Shapiro–Wilk test, where *p*-values above 0.05 confirm residual normality; the Box-Ljung test, where high *p*-values indicate the absence of significant autocorrelation; and the ARCH LM test, where high *p*-values suggest homoscedasticity, while low values reveal the presence of volatility clustering.

The ARIMA models exhibit varying levels of complexity and performance across the monitored gases. H_2_ and CH_4_ include drift or a non-zero mean, reflecting underlying trends, while C_2_H_2_ requires second-order differencing (*d* = 2), indicating greater non-stationarity. The Box–Cox transformation (*λ*) is close to 1 for H_2_, suggesting near-normal behavior, whereas CH_4_ has the lowest *λ* (0.07754), requiring a stronger transformation to stabilize the variance.

In terms of model fit, C_2_H_2_ achieves the highest log-likelihood (–31.22), indicating a better fit, while CH_4_ presents the lowest (–152.4), suggesting greater noise or structural shifts. Forecasting accuracy, assessed through RMSE, is lowest for CH_4_ (0.3077), indicating a more predictable trend, whereas C_2_H_4_ exhibits the highest RMSE (6.9917), reflecting greater uncertainty in predictions.

Residual diagnostics confirm that C_2_H_2_ has well-behaved residuals (ACF1 = −0.0123), while CH_4_ shows residual correlation (ACF1 = 0.2443). The Shapiro–Wilk test confirms normality for all gases except CH_4_, reinforcing the need for variance stabilization in methane forecasts. The Box-Ljung test indicates no significant autocorrelation, confirming that the models sufficiently capture temporal dependencies.

The ARCH LM test suggests mild heteroscedasticity for C_2_H_6_ (*p*-value = 0.04912) and C_2_H_4_ (*p*-value = 0.04526), though values remain above 0.01, making the models statistically valid but requiring careful interpretation. Overall, the models effectively capture gas concentration trends, supporting predictive analysis, though minor limitations in CH_4_ and variance instability in C_2_H_6_ and C_2_H_4_ should be considered in practical applications.

**Figure 4:**
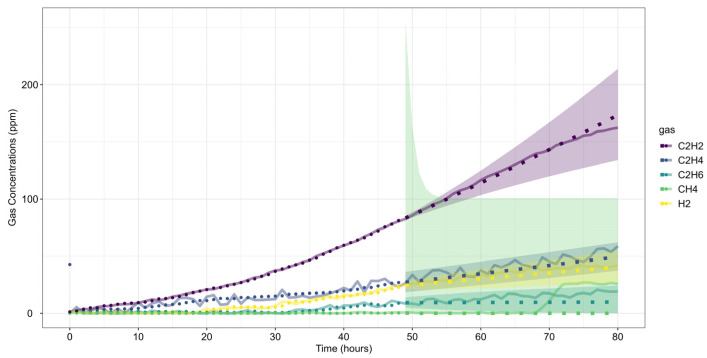
Case Study 1: ARIMA model data and projections on the original data.

Figure 4 presents the gas concentration forecasts generated by the ARIMA models. Dots represent the training data, small squares indicate the mean forecasts, and shaded areas denote the prediction intervals. The original dataset is shown as a solid line for comparison.

**Figure 5:**
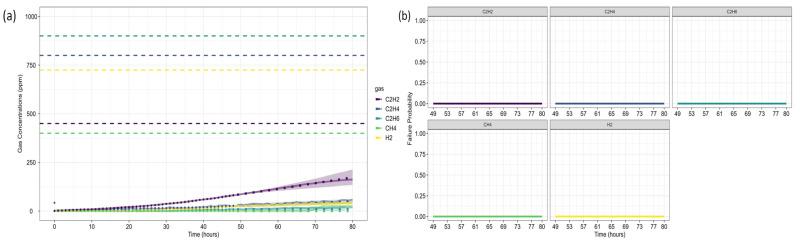
Case Study 1: ARIMA Forecasts and Equipment Failure Probability: (**a**) ARIMA forecasts with pre-failure thresholds for each gas (dashed lines); (**b**) Equipment failure probability based on each forecasted gas concentration.

**Figure 6:**
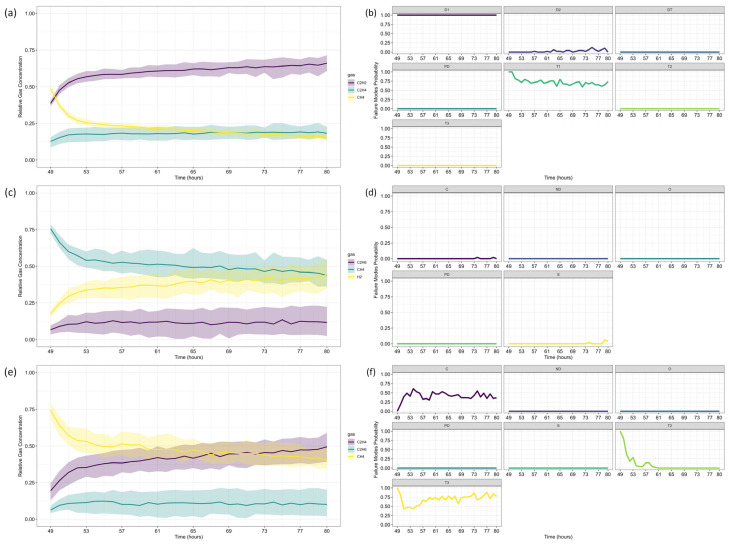
Case Study 1: Duval Triangle Analysis—Gas Projections and Failure Mode Probabilities: (**a**,**c**,**e**) Forecasted relative gas concentrations for Duval Triangles 1, 4, and 5 over time; (**b**,**d**,**f**) Probability of failure mode evolution for Duval Triangles 1, 4, and 5. The colored lines in (**b**,**d**,**f**) correspond to the respective failure modes indicated by the abbreviations in each panel title.

**Figure 7:**
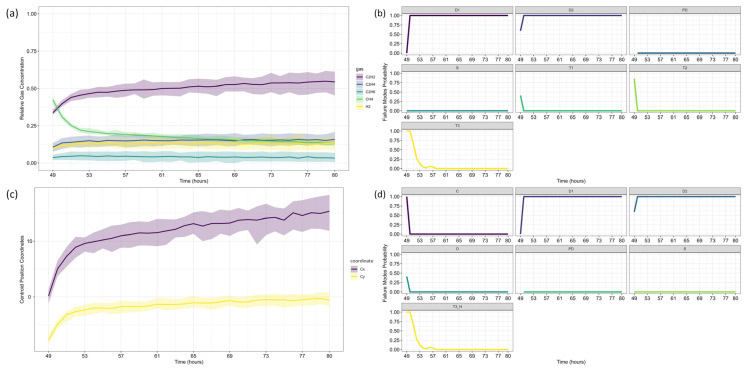
Case Study 1: Duval Pentagon Analysis—Gas Projections, Centroid Evolution, and Failure Mode Probabilities: (**a**) Forecasted relative gas concentrations over time; (**b**,**d**) Probability of failure mode evolution for Pentagons 1 and 2; (**c**) Centroid position evolution with x-y coordinates and uncertainties. The colored lines in (**b**,**d**) correspond to the respective failure modes indicated by the abbreviations in each panel title.

After forecasting, the analysis proceeds to Fault Prognosis, estimating the probability of failure within 32 h, and Fault Diagnosis, assessing fault type likelihood. Figure 5 displays the forecasted gas concentrations, failure thresholds, and associated probabilities. Since none of the gases exceed CIGRE’s pre-failure limits [15], the predicted failure probability is zero. Figure 6 and Figure 7 present the fault classification results using Duval’s Triangle and Pentagon analyses.

Duval Triangle 1 (Figure 6a,b) indicates that the D1 failure mode (low-energy electrical discharge) remains at 100% probability, while T1 (low-temperature thermal fault) also appears with a significant probability (>60%) throughout most of the forecast horizon. The probability of D2 (high-energy discharge) remains marginal, suggesting that a severe discharge is unlikely.

Given the presence of T1, further assessment using Duval Triangle 4 was conducted, but no substantial probability of fault evolution was detected (Figure 6c,d), reinforcing the absence of a developing thermal fault trend. Although Triangle 5 is not typically used in this scenario, its projections suggest a notable probability of T2 (intermediate-temperature thermal fault) and C (carbonization of insulation), as presented in Figure 6e,f.

Duval Pentagon 1 (Figure 7a–c) confirms D1 and D2 as dominant failure modes, both reaching 100% probability. Since Pentagon 1 alone is sufficient to establish a diagnosis in this scenario, Pentagon 2 (Figure 7a,b,d) serves only as a confirmation, rather than introducing new insights. According to best practices, similar failure types should be interpreted collectively, considering the hierarchical severity of failure modes. The most probable diagnosis is a D2 fault (high-energy discharge), followed by D1 (low-energy discharge), aligning with probabilistic trends across the different Duval methodologies.

The results are consistent with Riedmann (2023) [41], who observed that gas loss mechanisms in free-breathing transformers could alter fault classifications over time, impacting condition assessments. Their study highlighted a transition in Duval Triangle 1 from D1 to D2 after 57 h, reinforcing the importance of time-dependent dissolved gas diagnostics. The current analysis corroborates these findings, as D1 remains dominant throughout the forecast period, with T1 maintaining a high probability. However, unlike Riedmann’s study, no significant shift to D2 was detected within the analyzed time horizon when using triangles alone. The Pentagon analysis, however, suggests an early indication of D2, revealing a possible fault evolution trend not immediately apparent in Triangle-based assessments.

Additionally, Duval Triangle 4 (Figure 6c,d) does not indicate strong thermal fault evolution, countering the hypothesis that gas loss effects lead to misinterpretation of thermal faults due to differential degassing rates. The combined analysis of Triangle 4 and Pentagons suggests that the fault is more likely an evolving electrical arc rather than a developing thermal fault. While Triangle 5 indicates possible T2 and C faults, these results should not be considered in this case, following best-practice classification guidelines.

Riedmann [41] also emphasized that gas loss effects could distort Duval Triangle classifications, potentially masking failure modes. While the present study does not focus explicitly on gas loss corrections, the Pentagon-based fault evolution analysis confirms that D1 and D2 remain the most probable mechanisms, reinforcing the robustness of the classification approach.

This highlights a fundamental advantage of the proposed probabilistic framework: even in the presence of potential masking effects, the method successfully predicts fault evolution, quantifies uncertainties, and provides a comprehensive assessment of equipment health. Unlike deterministic approaches that classify faults solely based on observed data, this method enables early fault detection, enhances risk assessment, and supports proactive decision-making. However, its application requires careful evaluation by engineers and technical teams, as demonstrated in this case study.

### 4.2. Case Study 2: Thermal Fault in a 345 kV Power Transformer

This case study evaluates a thermal fault in a 345 kV power transformer, based DGA monitoring data, as reported by Riedmann (2022) [79]. The fault was initially detected through abnormal increases in C_2_H_4_ and CH_4_. Following the shutdown and internal inspection, carbonization of the paper insulation was confirmed.

**Figure 8:**
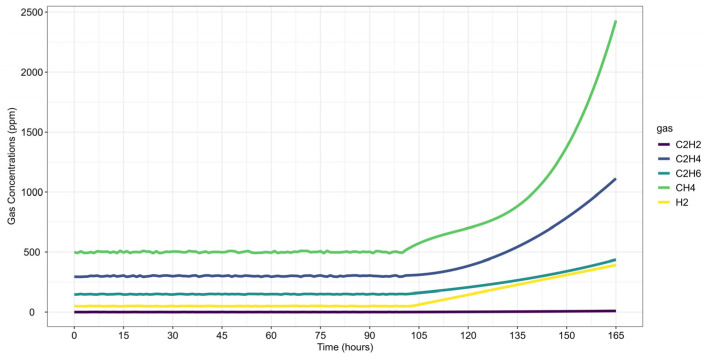
Case Study 2: Gas Concentrations Over Time.

Gas concentration data were collected over the last 165 h before shutdown (Figure 8). The preprocessed dataset follows the same concentration and rate-of-increase thresholds as in Case Study 1 (Table 4). The analysis window (*w*) includes 48 observations to maintain consistency and modeling stability, while the forecast window (*h*) spans 24 observations, representing a one-day horizon for short-term failure risk assessment.

CH_4_ was the first gas to exceed its detection threshold at time step 0, with fault confirmation occurring 103 h later. This delay is explained by CH_4_ concentrations already surpassing pre-failure thresholds from the first observed time step and remaining stable until approximately time step 100, when all gases except C_2_H_2_ showed a concentration increase.

With the fault confirmed, data forecasting was performed. Unlike the first case study, this analysis considers two reference time steps: 103 and 139, allowing for an additional evaluation 36 h after fault confirmation. This approach highlights the framework’s ability to track fault evolution over time.

Following the same methodology as Case Study 1, Table 6 and Table 7 present the ARIMA model parameters and residual diagnostics for each monitored gas at *τ* = 103 and *τ* = 139, respectively.

At time step 103, the ARIMA models applied to H_2_, C_2_H_6_, C_2_H_4_, and C_2_H_2_ follow a (0,0,0) structure with a non-zero mean, indicating that these gas concentrations remain relatively stable within this time window. In contrast, CH_4_ follows a (1,1,0) model, incorporating a first-order autoregressive term and differencing, suggesting some temporal dependency.

Variance stabilization, assessed via Box–Cox *λ* varies significantly across gases. C_2_H_6_ and C_2_H_4_ have *λ* = 1.9999, suggesting that the original data required minimal transformation. Conversely, CH_4_ and H_2_ have *λ* = −0.8999, indicating the need for stronger variance adjustments.

In terms of model fit, CH_4_ achieves the highest log-likelihood (399.41), suggesting a good representation of its concentration dynamics, while C_2_H_4_ (–414.05) presents the lowest value, indicating a weaker model fit. RMSE values confirm that CH_4_ exhibits the highest forecasting error (17.9064), whereas C_2_H_2_ achieves the lowest (0.3304).

Residual diagnostics reveal mixed results. ACF1 values indicate strong autocorrelation in H_2_ (0.8160) and C_2_H_4_ (0.8359), suggesting some remaining temporal dependencies. Normality tests confirm that C_2_H_6_ is the only gas with normally distributed residuals, while all others deviate from normality.

The Box-Ljung test confirms that all models adequately capture residual dependencies, with *p*-values above 0.05, indicating no significant autocorrelation. However, the ARCH LM test identifies heteroscedasticity in CH_4_ (*p*-value = 0.0092), suggesting variance instability (which may impact long-term forecasting reliability), while other gases maintain stable variance.

At time step 139, the models show increased complexity, incorporating higher-order differencing and autoregressive components, reflecting evolving gas concentration dynamics. All gases require second-order differencing (*d* = 2), emphasizing a stronger non-stationarity compared to *τ* = 103.

Box–Cox *λ* values shift, with H_2_ (0.7301) and CH_4_ (0.3797) requiring moderate variance transformations, whereas C_2_H_2_ (1.6785) is closer to normality. C_2_H_4_ (−0.2405) exhibits a more extreme transformation need, suggesting greater variance instability.

In terms of model fit, C_2_H_4_ achieves the highest log-likelihood (231.76), while H_2_ presents the lowest (–20.48). RMSE values indicate CH_4_ (5.5989) maintains a higher error rate, although reduced compared to *τ* = 103, while C_2_H_2_ (0.1683) exhibits the lowest error.

Residual diagnostics show a slight reduction in autocorrelation compared to the earlier reference point, with ACF1 values stabilizing. However, normality tests indicate that none of the gases follow a normal distribution, reinforcing the need for further variance stabilization.

The Box-Ljung test suggests adequate residual independence for most gases, except for C_2_H_4_ (*p*-value = 0.02489), indicating possible autocorrelation effects. Additionally, the ARCH LM test detects heteroscedasticity in C_2_H_6_ (*p*-value = 0.02719) and C_2_H_4_ (*p*-value = 9.944 × 10^−7^), indicating significant variance instability.

Overall, the results indicate that gas concentration trends evolve, requiring more complex ARIMA models and greater differencing at *τ* = 139. The improvement in RMSE for CH_4_ suggests a better adaptation in the time-series behavior. However, the increase in heteroscedasticity and residual dependencies at later time steps emphasizes the importance of uncertainty quantification in predictive maintenance strategies.

**Figure 9:**
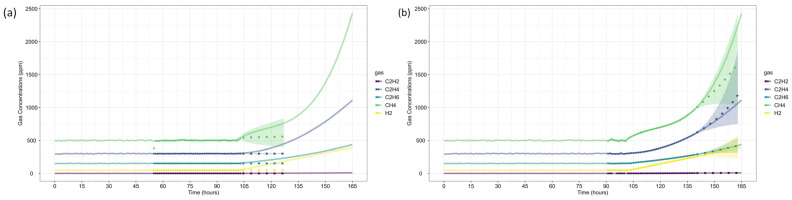
Case Study 2: ARIMA Model Data and Forecasts: (**a**) Forecasts at *τ* = 103; (**b**) Forecasts at *τ* = 139.

Following the same methodology as before, data forecasting was performed at two reference time steps (*τ* = 103 and *τ* = 139), allowing for a comparative assessment of forecast evolution over time. The ARIMA model predictions for each gas are shown in Figure 9, where dots represent training data, small squares indicate mean forecasts, and shaded areas denote confidence intervals. The original dataset is displayed as a solid line for reference, with (a) corresponding to *τ* = 103 and (b) to *τ* = 139.

**Figure 10:**
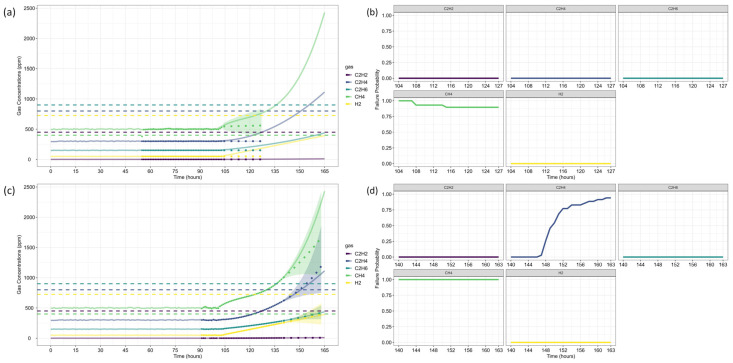
Case Study 2: ARIMA Forecasts and Transformer Failure Probability: (**a**,**c**) Forecasted gas concentrations and pre-failure thresholds at *τ* = 103 and *τ* = 139 (dashed lines); (**b**,**d**) Transformer failure probability based on each gas concentration threshold at *τ* = 103 and *τ* = 139.

Next, the analysis proceeded to Fault Prognosis, estimating the probability of transformer failure at both reference time steps. Figure 10 presents the forecasted values and pre-failure thresholds for each gas, along with the computed failure probability over the *h*-window (24 h). A key observation in this case study is that CH_4_ concentrations exceeded the pre-failure threshold from the first recorded time step. Consequently, at *τ* = 103, the failure probability was already close to 100%, primarily driven by CH_4_. By *τ* = 139, C_2_H_4_ concentrations also increased significantly, triggering a failure condition due to both gases simultaneously.

**Figure 11:**
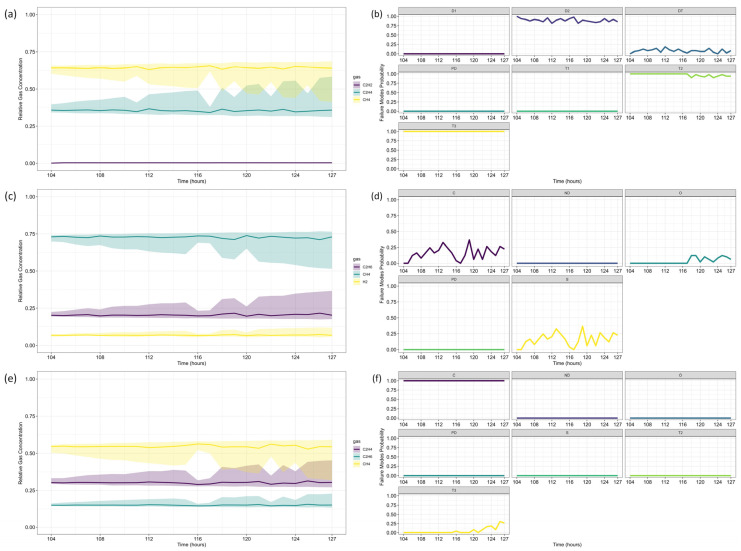
Case Study 2, *τ* = 103: Duval Triangle Analysis—Gas Projections and Failure Mode Probabilities: (**a**,**c**,**e**) Forecasted relative gas concentrations for Duval Triangles 1, 4, and 5 over time; (**b**,**d**,**f**) Probability of failure mode evolution for Duval Triangles 1, 4, and 5. The colored lines in (**b**,**d**,**f**) correspond to the respective failure modes indicated by the abbreviations in each panel title.

**Figure 12:**
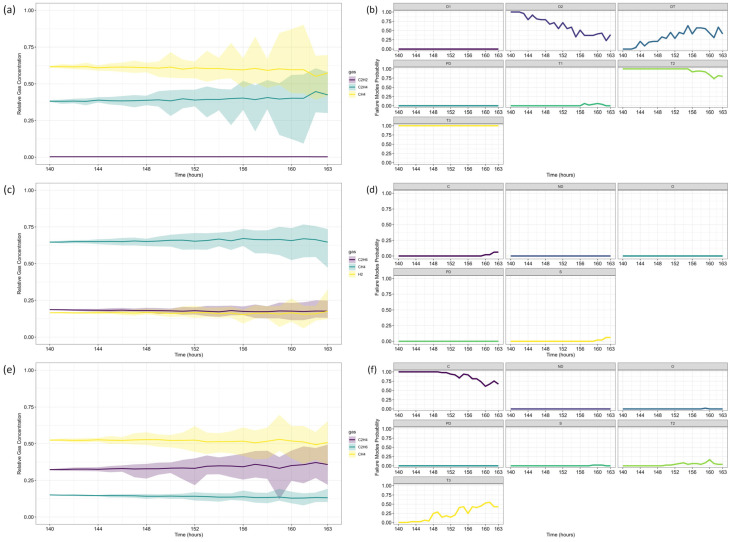
Case Study 2, *τ* = 139: Duval Triangle Analysis—Gas Projections and Failure Mode Probabilities: (**a**,**c**,**e**) Forecasted relative gas concentrations for Duval Triangles 1, 4, and 5 over time; (**b**,**d**,**f**) Probability of failure mode evolution for Duval Triangles 1, 4, and 5. The colored lines in (**b**,**d**,**f**) correspond to the respective failure modes indicated by the abbreviations in each panel title.

**Figure 13:**
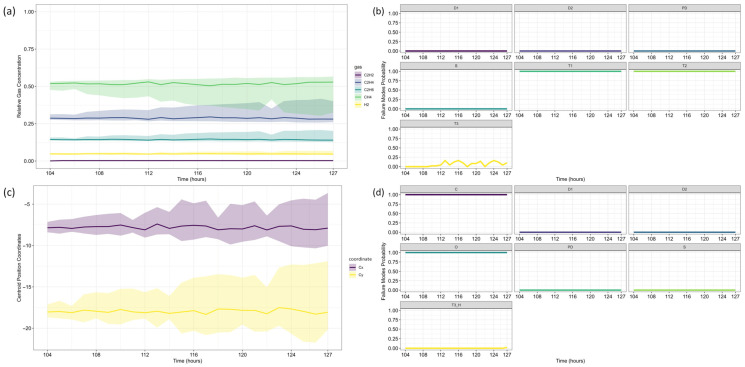
Case Study 2, *τ* = 103: Duval Pentagon Analysis—Gas Projections, Centroid Evolution, and Failure Mode Probabilities: (**a**) Forecasted relative gas concentrations over time; (**b**,**d**) Probability of failure mode evolution for Pentagons 1 and 2; (**c**) Centroid position evolution with x-y coordinates and uncertainties. The colored lines in (**b**,**d**) correspond to the respective failure modes indicated by the abbreviations in each panel title.

**Figure 14:**
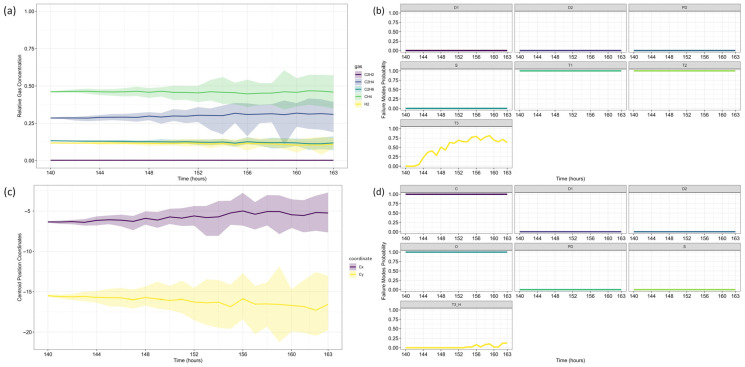
Case Study 2, *τ* = 139: Duval Pentagon Analysis—Gas Projections, Centroid Evolution, and Failure Mode Probabilities: (**a**) Forecasted relative gas concentrations over time; (**b**,**d**) Probability of failure mode evolution for Pentagons 1 and 2; (**c**) Centroid position evolution with x-y coordinates and uncertainties. The colored lines in (**b**,**d**) correspond to the respective failure modes indicated by the abbreviations in each panel title.

For Fault Diagnosis, the Duval Triangle and Pentagon analyses were conducted at both reference time steps, identifying the evolution of failure modes over time. The Duval Triangle analysis results are presented in Figure 11 and Figure 12, corresponding to *τ* = 103 and *τ* = 139, respectively. Similarly, the Duval Pentagon analysis results are detailed in Figure 13 and Figure 14 for the same time steps.

The probabilistic analysis of Duval’s Triangles and Pentagons in the second case study reveals key insights into the progression of transformer faults over time, demonstrating how failure modes evolve dynamically rather than remaining fixed as in deterministic approaches. By quantifying uncertainty and interdependencies, this framework offers a more comprehensive diagnostic perspective compared to traditional methods.

At *τ* = 103, Duval Triangle 1 (Figure 11a,b) identifies D2 (high-energy electrical discharge) as the dominant fault mode (~100% probability), with DT (combined electrical and thermal faults) appearing at lower probability levels. Simultaneously, T2 (thermal fault between 300–700 °C) is detected at 100% probability until *t* = 116, after which T3 (thermal fault > 700 °C) becomes predominant throughout the forecast horizon. This trend indicates a progressive increase in thermal severity, consistent with insulation material degradation under sustained overheating conditions.

Given the strong presence of T3, further evaluation using Duval Triangles 4 and 5 was conducted. Triangle 4 (Figure 11c,d) suggests a high probability of C (carbonization of paper) and S (gas loss from mineral oil), with a minor probability of O (overheating < 250 °C) emerging at *t* = 116. Triangle 5 (Figure 11e,f) further reinforces this trend, maintaining a 100% probability of C throughout the entire period, while T3 exhibits a clear increasing trend from *t* = 116 onward. These findings confirm progressive thermal degradation leading to paper carbonization, suggesting long-term structural damage to the insulation system.

The Duval Pentagon analysis at *τ* = 103 supports these conclusions. Pentagon 1 (Figure 13a–c) confirms a 100% probability for T1 (thermal fault < 300 °C) and T2, with T3 appearing at lower probability levels, indicating thermal stress escalation. Meanwhile, Pentagon 2 (Figure 13a,b,d) shows 100% probability for both C and O, reflecting severe degradation of both insulation materials and oil stability.

By *τ* = 139, notable shifts in fault probabilities emerge (Figure 12b). D2 probability declines, while DT increases slightly, suggesting a gradual transition in fault characteristics. However, T2 and T3 remain stable, confirming ongoing thermal degradation mechanisms. At this stage, Triangle 4 (Figure 12c,d) no longer indicates significant probabilities for additional failure types, while Triangle 5 (Figure 12e,f) maintains 100% probability for C until *t* = 152, after which a slight decline occurs, coinciding with a rising probability of T3. This pattern suggests that while carbonization remains the primary failure mode, severe thermal degradation (T3) continues to intensify.

The Duval Pentagon results at *τ* = 139 reinforce these observations (Figure 14). Pentagon 1 still confirms 100% probability for T1 and T2, but T3 experiences a significant rise, indicating increasing thermal stress and insulation deterioration. Meanwhile, Pentagon 2 maintains 100% probability for C and O, with T3-H (thermal fault affecting oil only) emerging from *t* = 152 onward, signaling progressive oil degradation as an additional failure mechanism.

These results align with Riedmann (2022) [79], which classified the observed failure as a thermal fault confirmed by post-shutdown inspection, revealing carbonization of paper insulation. The current study corroborates this conclusion, as the probabilistic approach successfully predicts fault progression (T1 → T2 → T3), capturing insulation degradation trends over time.

A key distinction between the proposed method and deterministic approaches lies in its ability to forecast fault evolution and quantify uncertainty. While Riedmann’s analysis relied on post-failure assessments, the probabilistic framework anticipates insulation degradation trends and the increasing probability of severe faults before transformer shutdown. This capability is crucial for early maintenance planning, enabling proactive risk mitigation instead of reactive fault detection.

As seen in the gas concentration forecasts, uncertainty increases over time, leading to greater variability in fault probability estimates. This effect is particularly pronounced in longer forecast horizons, where fault probabilities fluctuate due to cumulative model uncertainties and increased gas concentration dispersion. The residual behavior observed in Table 6 and Table 7, particularly the presence of heteroscedasticity and autocorrelation, reinforces this observation, as indicated by the ARCH LM test results confirming time-dependent variance for certain gases. These findings highlight the importance of carefully interpreting long-term predictions, recognizing that model confidence decreases as the forecast horizon extends.

Despite this, the probabilistic approach remains superior to deterministic methods, as it enables a dynamic assessment of evolving failure risks rather than relying on fixed classification thresholds. By quantifying uncertainty, this framework provides a more realistic representation of transformer conditions, ensuring that maintenance decisions incorporate both fault likelihoods and confidence levels in those assessments.

### 4.3. Model Performance and Predictive Reliability

The performance of the proposed probabilistic framework was evaluated through two documented case studies drawn from the literature, which serve as validated benchmarks for fault diagnosis and gas behavior in power transformers. These benchmarks, presented by Riedmann et al. [41,79], offer well-characterized scenarios involving thermal degradation and controlled electrical discharges under varying operational conditions.

In both case studies, the framework achieved 100% accuracy in fault type classification when compared to the diagnoses originally reported by Riedmann. Moreover, all observed gas concentrations fell within the 95% confidence intervals generated by the ARIMA-based forecasts, as illustrated in Figure 4 and Figure 10. This confirms that the models provide statistically consistent and reliable predictions, even in the presence of nonstationarity and short time series.

To evaluate forecast performance beyond visual agreement, residual diagnostics were conducted to assess the quality and stability of the ARIMA models. While some gases showed signs of autocorrelation and variance instability, especially at later forecast horizons, the models still captured the overall trends effectively. The presence of residual autocorrelation and variance instability observed in specific cases suggests that some gases exhibit more complex temporal behaviors as faults evolve.

Still, the forecast envelopes proved resilient, preserving predictive reliability even under departures from ideal statistical conditions. This indicates that the proposed framework provides robust forecasts with well-calibrated uncertainty bounds. It remains effective even when statistical assumptions are partially violated, offering a practical and interpretable tool for supporting early fault detection and maintenance planning.

An additional strength of the proposed approach is its capacity to anticipate fault behavior well before the end of the observation period, a critical feature for operational decision-making in asset management. In the first case study, derived from Riedmann (2023) [41], data were available up to time step *t* = 80, but the full diagnostic and prognostic analysis was performed at *t* = 48. This implies a 32-step look-ahead window, during which the framework maintained consistent classification of the D1 fault mode and detected early probabilistic signals of D2 escalation.

Notably, Riedmann’s own analysis observed a transition from D1 to D2 only after 57 h, reinforcing the importance of time-dependent DGA interpretation and highlighting the benefit of early detection. The proposed method was thus able to identify the dominant failure mode and anticipate a potential escalation earlier than the transition point reported in the benchmark study.

In the second case study, based on Riedmann (2022) [79], measurements extended up to *t* = 163, but the model conducted diagnostic assessments at two earlier points: *τ* = 103 and *τ* = 139. These points were chosen to evaluate the progression of thermal degradation, and the framework successfully identified the evolution from T1 to T3 faults, along with early signs of carbonization (C) and oil degradation (O), well before the final shutdown and post-failure inspection.

The prediction at *τ* = 103 revealed early indicators of severe thermal behavior, while the update at *τ* = 139 confirmed the intensification of degradation mechanisms and the emergence of oil-related fault signatures. This anticipatory capability supports risk-based maintenance strategies and aligns with IEC and IEEE recommendations on continuous monitoring and early intervention.

The diagnostic module integrates probabilistic versions of Duval’s Triangles and Pentagons, which are widely accepted tools in transformer fault analysis. By replacing hard boundaries with probabilistic classification zones, the framework accounts for both measurement uncertainty and overlapping fault signatures.

This is particularly relevant in ambiguous conditions, such as partial discharges co-occurring with thermal effects, or scenarios affected by differential gas loss rates, where deterministic classification may oscillate or fail to produce consistent results. In contrast, the probabilistic model maintains stability by distributing fault likelihood across multiple potential classes and tracking their evolution over time.

## 5. Conclusions

This study introduced a probabilistic framework for transformer fault detection, diagnosis, and risk-based prognosis, integrating self-adaptive ARIMA modeling with an uncertainty-aware extension of Duval’s methods. By incorporating probabilistic fault classification and time-series forecasting of dissolved gas concentrations, the proposed approach provides a more adaptive, data-driven, and computationally efficient alternative to deterministic and machine learning-based methods for DGA-based transformer analysis.

It should be noted that the proposed forecasting component estimates the probabilistic evolution of measured dissolved gas concentrations, rather than attempting to predict specific fault types deterministically. While fault mechanisms in transformers may change or appear intermittently, the gas dynamics themselves exhibit consistent temporal correlations that can be statistically modeled and used to infer fault-related risk under uncertainty.

To validate the proposed method, two case studies were conducted, demonstrating its ability to forecast the individual behavior of dissolved gases, predict their concentration trends, estimate fault evolution, and provide dynamic risk assessments.

The first case study, based on an experimental oil tank setup, highlighted the model’s capability to forecast gas concentration trends and estimate fault evolution, even under gas loss conditions that often distort traditional DGA-based classifications. The probabilistic adaptation of Duval’s Triangle and Pentagon enabled a refined failure mode interpretation, distinguishing low-energy (D1) and high-energy (D2) discharges while mitigating gas diffusion effects.

The second case study, involving a 345 kV power transformer with a thermal fault, further validated the model’s effectiveness. The proposed framework successfully tracked the progressive thermal degradation (T1 → T2 → T3), forecasting increasing failure probabilities before the transformer was taken offline. Unlike conventional deterministic methods that rely on fixed thresholds, this probabilistic approach enables continuous risk assessment and supports earlier, more proactive maintenance decisions.

A key strength of the proposed framework lies in its ability to quantify uncertainty in both fault classification and prognosis while preserving structured diagnostic inference through established DGA methodologies, such as Duval’s polygons. This addresses a major limitation of traditional DGA techniques, which rely on fixed thresholds and lack adaptability. Besides, the ARIMA-based modeling approach eliminates the need for labeled datasets, offering a scalable and practical solution for real-world transformer monitoring based on continuous DGA sensor data. Its probabilistic design enhances adaptability, enabling dynamic updates of forecasts and failure probabilities in response to evolving gas behavior.

Another important feature of the framework is its ability to accommodate the specific nature of different fault types. Because each gas is modeled individually, the method naturally captures both fast-changing dynamics, such as intermittent partial discharges, and slower progressions, such as thermal degradation. The subsequent probabilistic reformulation of Duval’s polygons ensures that these distinct gas signatures are consistently reflected in the diagnostic stage, further strengthening the robustness and applicability of the approach.

Future research could focus on hybrid models integrating ARIMA with nonlinear techniques, enhancing adaptability while preserving interpretability, thereby further improving transformer fault prognosis. Expanding the model to account for varying operational conditions, such as seasonal fluctuations and sudden load changes, would further strengthen its robustness under real-world conditions. Finally, implementing the framework in real-time continuous monitoring systems, particularly those enabled by online DGA sensors and IoT-based infrastructures, would provide a definitive validation of its industrial applicability, bridging the gap between theoretical advancements and practical reliability in power system maintenance.

Another promising direction is to explicitly account for the influence of operating temperature and load on gas solubility, either by incorporating these variables as exogenous inputs in the forecasting models or by adjusting concentrations according to known solubility curves, thereby reducing uncertainty in the presence of daily fluctuations. Future work may also address the recently introduced fault subtypes, including the new sub-zones in Duval’s Triangles and Pentagons that distinguish arcing in oil from arcing in paper, as well as additional categories related to paper insulation carbonization. Although not yet consolidated in current standards, their eventual integration into the proposed framework could further improve diagnostic resolution and align the methodology with emerging advances in DGA interpretation.

The findings presented demonstrate that the proposed probabilistic approach represents a significant advancement in DGA-based transformer fault analysis, offering improved predictability, robustness, and reliability for risk-based decision-making. By incorporating uncertainty quantification and dynamic forecasting, the framework contributes to a scalable and interpretable PHM solution tailored to critical power assets. Its alignment with industry standards and adaptability to real-world conditions ensure practical relevance for enhancing transformer reliability within modern power systems, sensor-enabled monitoring environments, and smart grids.

## Figures and Tables

**Table 1:** Transformer Failure Modes (basic faults and fault subtypes) Detectable via DGA.

Code	Fault Type	Description
R	Catalytic Reactions	Occur due to moisture and interactions with galvanized steel in transformer oil sampling valves or rust on the tank steel.
PD	Partial Discharges	Formation of cold plasma discharges, potentially leading to wax-like deposits on the insulating paper.
S	Stray gassing from Mineral Oil	Occurs at temperatures below 200 °C due to chemical instability caused by modern refining techniques or incompatibility between materials (metal passivators).
T1	Thermal Failure < 300 °C	Occurs in mineral oil and/or paper due to temperatures below 300 °C, causing paper discoloration (browning).
O	Overheating < 250 °C	Affects paper or mineral oil at temperatures below 250 °C, without carbonization of the paper or loss of its electrical insulation properties.
C	Carbonization of Paper	Possible carbonization of paper insulation, indicating severe overheating or prolonged thermal stress, typically associated with T2–T3 thermal ranges (>300 °C).
T2	Thermal Failure 300–700 °C	Occurs in paper insulation, leading to burning when exposed to temperatures between 300 °C and 700 °C.
T3	Thermal Failure > 700 °C	Severe thermal degradation, with strong evidence of mineral oil carbonization, metal discoloration (~800 °C), or metal melting (>1000 °C).
T3-H	Thermal Failure (Oil only, >700 °C)	Specific to mineral oil, involving extensive degradation without significant damage to solid insulation.
D1	Low-Energy Discharges	Partial spark-type discharges in mineral oil and/or paper, causing charred perforations in insulation, surface carbonization traces, and free carbon particles in the oil. Often associated with divergent tap-changer operation.
D2	High-Energy Discharges	Occur in mineral oil and/or paper, evidenced by extensive destruction and carbonization of paper, metal melting at discharge ends, and severe carbonization of oil. In some cases, this leads to equipment shutdown, confirming the presence of large current flow.
DT	Combined Electrical and Thermal Faults	Represents intermediate fault conditions, where both electrical discharges and thermal failures coexist within the transformer.
N	Not Detected/ Not Identified	Indicates that no fault has been classified based on the available dissolved gas concentrations. However, this does not confirm that the transformer is in a normal operating condition, as early-stage faults or transient issues may not yet be detectable.

**Table 2:** Transformer Failure Modes Detectable via DGA.

Method	Handles Autocorrelation?	Works with Few Data Points?	Interpretability	Computational Cost	Suitability for DGA
ARIMA	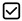 Yes	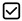 Yes	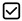 High	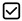 Low	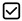 Best fit
Holt-Winters	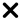 No	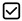 Yes	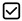 High	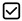 Low	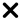 Lacks autocorrelation modeling
BSTS	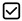 Yes	◇ Moderate	◇ Moderate	◇ Moderate	◇ Possible alternative
GPR	◇ Partial	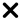 No	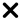 Low	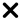 High	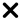 Not feasible
LSTM/GRU	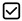 Yes	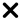 No	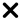 Low	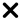 High	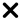 Not feasible
Transformers	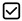 Yes	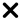 No	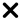 Low	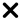 Very high	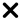 Not feasible

**Table 3:** Angular Positions and Indexes of Gases in Duval’s Pentagon.

Gas	Symbol	*θ*_g_ (Degrees)	Index *i*
Hydrogen	H_2_	90°	1
Ethane	C_2_H_6_	162°	2
Methane	CH_4_	234°	3
Ethylene	C_2_H_4_	306°	4
Acetylene	C_2_H_2_	18°	5

**Table 4:** Detection, Confirmation, and Failure Prognosis Thresholds.

Gas	Symbol	Detection Threshold	Confirmation Threshold	Failure Threshold
Hydrogen	H_2_	100 ppm	179 ppm/year	725 ppm
Ethane	C_2_H_6_	55 ppm	175 ppm/year	400 ppm
Methane	CH_4_	80 ppm	176 ppm/year	900 ppm
Ethylene	C_2_H_4_	170 ppm	218 ppm/year	800 ppm
Acetylene	C_2_H_2_	3 ppm	7 ppm/year	450 ppm

**Table 5:** Case Study 1: ARIMA Model Parameters and Residual Diagnostic Test Results.

Gas	H_2_	C_2_H_6_	CH_4_	C_2_H_4_	C_2_H_2_
Model (*p*, *d*, *q*)	(1,1,0) with drift	(0,1,1)	(1,0,0) with non-zero mean	(0,1,1) with drift	(2,2,1)
Box–Cox *λ*	0.9308	0.7332	0.07754	0.7479	0.8410
Log-Likelihood	–82.8	–79.19	–152.4	–100.87	–31.22
RMSE	1.4873	1.3989	0.3077	6.9917	0.7250
ACF1	−0.0666	0.1007	0.2443	–0.0695	–0.0123
Normality	Normal	Normal	Not Normal	Normal	Normal
Box-Ljung *p*-value	0.3832	0.451	0.6331	0.8144	0.419
ARCH LM *p*-value	0.6749	0.04912	0.9523	0.04526	0.8858

**Table 6:** Case Study 2: ARIMA Model Parameters and Residual Diagnostic Test Results (*t* = 103).

Gas	H_2_	C_2_H_6_	CH_4_	C_2_H_4_	C_2_H_2_
Model (*p*, *d*, *q*)	(0,0,0) with non-zero mean	(0,0,0) with non-zero mean	(1,1,0)	(0,0,0) with non-zero mean	(0,0,1) with non-zero mean
Box–Cox *λ*	–0.8999	1.9999	–0.8999	1.9999	0.1886
Log-Likelihood	285.79	–342.64	399.41	–414.05	–109.07
RMSE	1.2029	1.7505	17.9064	3.7658	0.3304
ACF1	0.8160	–0.1463	0.0076	0.8359	0.2600
Normality	Not Normal	Normal	Not Normal	Not Normal	Not Normal
Box-Ljung *p*-value	0.8681	0.481	0.9849	0.662	0.1898
ARCH LM *p*-value	0.6605	0.6375	0.009239	0.7116	0.5765

**Table 7:** Case Study 2: ARIMA Model Parameters and Residual Diagnostic Test Results (*t* = 139).

Gas	H_2_	C_2_H_6_	CH_4_	C_2_H_4_	C_2_H_2_
Model (*p*, *d*, *q*)	(1,2,0)	(2,2,0)	(0,2,1)	(2,2,2)	(1,2,1)
Box–Cox *λ*	0.7301	0.1776	0.3797	–0.2405	1.6785
Log-Likelihood	–20.48	122.73	33.31	231.76	39.02
RMSE	1.0617	1.0529	5.5989	2.1250	0.1683
ACF1	0.0017	0.1654	0.2322	–0.1441	–0.3428
Normality	Not Normal	Not Normal	Not Normal	Not Normal	Not Normal
Box-Ljung *p*-value	0.7589	0.1637	0.3936	0.02489	0.05745
ARCH LM *p*-value	0.03765	0.02719	0.4908	9.944 × 10^−7^	9.926 × 10^−7^

## Data Availability

Some or all the raw data supporting the findings of this study are available from the corresponding author upon reasonable request.

## Appendix A

This appendix provides the complete formulation of each failure mode probability expression considering Duval’s Triangle and Pentagon methods.

### Appendix A.1. Duval’s Triangle 1 (T1)

(A1)
$$P_{D1,t}=P\left(R_{C_{2}H_{4},t}<23,\;\;R_{C_{2}H_{2},t}>13\right)$$

(A2)
$$P_{D2,t}=P\left(23<R_{C_{2}H_{4},t}<40,\;\;13<R_{C_{2}H_{2},t}<29\right)+P\left(R_{C_{2}H_{4},t}>23,\;\;R_{C_{2}H_{2},t}>29\right)$$

(A3)
$$\begin{array}{c} P_{\mathrm{DT},t}=P\left(R_{C_{2}H_{4},t}<50,\;\;4<R_{C_{2}H_{2},t}<13\right)+\\ P\left(40<R_{C_{2}H_{4},t}<50,\;\;13<R_{C_{2}H_{2},t}<29\right)+\\ P\left(R_{C_{2}H_{4},t}>50,\;\;15<R_{C_{2}H_{2},t}<29\right) \end{array}$$

(A4)
$$P_{T1,t}=P\left(R_{{\mathrm{CH}}_{4},t}<98,\;\;R_{C_{2}H_{4},t}<20,\;R_{C_{2}H_{2},t}<4\right)$$

(A5)
$$P_{T2,t}=P\left(20<\;R_{C_{2}H_{4},t}<50,\;R_{C_{2}H_{2},t}<4\right)$$

(A6)
$$P_{T3,t}=P\left(R_{C_{2}H_{4},t}>50,\;R_{C_{2}H_{2},t}<15\right)$$

(A7)
$$P_{\mathrm{PD},t}=P\left(R_{{\mathrm{CH}}_{4},t}>98\right)$$

### Appendix A.2. Duval’s Triangle 2 (T2)

(A8)
$$P_{O,t}=P\left(R_{H_{2},t}<9,\;\;R_{C_{2}H_{6},t}>30\right)$$

(A9)
$$P_{C,t}=P\left(R_{{\mathrm{CH}}_{4},t}>36,\;\;R_{C_{2}H_{6},t}>24\right)+P\left(R_{H_{2},t}<15,\;24<\;R_{C_{2}H_{6},t}<29\right)$$

(A10)
$$\begin{array}{c} P_{S,t}=P\left(R_{H_{2},t}>9,\;\;30<R_{C_{2}H_{6},t}<46\right)+P\left(R_{H_{2},t}>15,\;24<R_{C_{2}H_{6},t}<30\right)+\\ P\left(R_{{\mathrm{CH}}_{4},t}<36,\;1<R_{C_{2}H_{6},t}<24\right)+P\left(15<R_{{\mathrm{CH}}_{4},t}<36,\;R_{C_{2}H_{6},t}<1\right)+\\ P\left(R_{{\mathrm{CH}}_{4},t}<2,\;R_{C_{2}H_{6},t}<1\right) \end{array}$$

(A11)
$$P_{\mathrm{PD},t}=P\left(2<R_{{\mathrm{CH}}_{4},t}<15,\;\;R_{C_{2}H_{6},t}<1\right)$$

(A12)
$$P_{\mathrm{ND},t}=P\left(\;R_{H_{2},t}>9,\;R_{C_{2}H_{6},t}>46\right)$$

### Appendix A.3. Duval’s Triangle 3 (T3)

(A13)
$$\begin{array}{c} P_{O,t}=P\left(1<R_{C_{2}H_{4},t}<10,\;2<R_{C_{2}H_{6},t}<14\right)+P\left(R_{C_{2}H_{4},t}<1,\;R_{C_{2}H_{6},t}<2\right)\\ +P\left(R_{C_{2}H_{4},t}<10,\;R_{C_{2}H_{6},t}>54\right) \end{array}$$

(A14)
$$P_{T2,t}=P\left(10<R_{C_{2}H_{4},t}<35,\;\;R_{C_{2}H_{6},t}<12\right)$$

(A15)
$$\begin{array}{c} P_{T3,t}=P\left(R_{C_{2}H_{4},t}>35,\;R_{C_{2}H_{6},t}<12\right)+P\left(R_{{\mathrm{CH}}_{4},t}>50,\;12<R_{C_{2}H_{6},t}<14\right)+\\ P\left(R_{C_{2}H_{4},t}>70,\;R_{C_{2}H_{6},t}<14\right)+P\left(R_{C_{2}H_{4},t}>35,\;R_{C_{2}H_{6},t}>30\right) \end{array}$$

(A16)
$$P_{\mathrm{PD},t}=P\left(R_{C_{2}H_{4},t}<1,\;2<R_{C_{2}H_{6},t}<14\right)$$

(A17)
$$P_{\mathrm{ND},t}=P\left(\;10<R_{C_{2}H_{4},t}<35,\;R_{C_{2}H_{6},t}>30\right)$$

(A18)
$$P_{S,t}=P\left(\;R_{C_{2}H_{4},t}<10,\;14<R_{C_{2}H_{6},t}<54\right)$$

(A19)
$$\begin{array}{c} P_{C,t}=P\left(\;10<R_{C_{2}H_{4},t}<50,\;12<R_{C_{2}H_{6},t}<14\right)+\\ P\left(\;10<R_{C_{2}H_{4},t}<70,\;14<R_{C_{2}H_{6},t}<30\right) \end{array}$$

### Appendix A.4. Duval’s Pentagon 1 (P1)

(A20) $$P_{\mathrm{PD},t}=P\left(\left(C_{x,t},C_{y,t}\right)\in R_{\mathrm{PD}}\right)$$

where

(A21) $$R_{\mathrm{PD}}=\left\{\left(0,\;33\right),\left(-1,\;33\right),\left(-1,\;24.5\right),\left(0,\;24.5\right)\right\}$$

(A22) $$P_{D1,t}=P\left(\left(C_{x,t},C_{y,t}\right)\in R_{D1}\right)$$

where

(A23) $$R_{D1}=\left\{\left(0,\;40\right),\left(38,\;12\right),\left(32,\;-6.1\right),\left(4,\;16\right),\left(0,\;1.5\right)\right\}$$

(A24) $$P_{D2,t}=P\left(\left(C_{x,t},C_{y,t}\right)\in R_{D2}\right)$$

where

(A25) $$R_{D2}=\left\{\left(4,\;16\right),\left(32,\;-6.1\right),\left(24.3,\;-30\right),\left(0,\;-3\right),\left(0,\;1.5\right)\right\}$$

(A26) $$P_{T1,t}=P\left(\left(C_{x,t},C_{y,t}\right)\in R_{T1}\right)$$

where

(A27) $$\begin{array}{c} R_{T1}=\left(-6,\;-4\right),\left(-22.5,\;-32.4\right),\left(-23.5,\;-32.4\right),\\ \left.\left(-35,\;3\right),\left(0,\;1.5\right),\left(0,\;-3\right)\right\} \end{array}$$

(A28) $$P_{T2,t}=P\left(\left(C_{x,t},C_{y,t}\right)\in R_{T2}\right)$$

where

(A29) $$R_{T2}=\left\{\left(-6,\;-4\right),\left(1,\;-32.4\right),\left(-22.5,\;-32.4\right)\right\}$$

(A30) $$P_{T3,t}=P\left(\left(C_{x,t},C_{y,t}\right)\in R_{T3}\right)$$

where

(A31) $$R_{T3}=\left\{\left(0,\;-3\right),\left(24.3,\;-30\right),\left(23.5,\;-32.4\right),\left(1,\;-32\right),\left(-6,\;-4\right)\right\}$$

(A32) $$P_{S,t}=P\left(\left(C_{x,t},C_{y,t}\right)\in R_{S}\right)$$

where

(A33) $$\begin{array}{c} R_{S}=\left\{\left(0,\;1.5\right),\left(-35,\;3.1\right),\left(-38,\;12.4\right),\left(0,\;40\right),\right.\left(0,\;33\right),\\ \left.\left(-1,\;33\right),\left(-1,\;24.5\right),\left(0,\;24.5\right)\right\} \end{array}$$

### Appendix A.5. Duval’s Pentagon 2 (P2)

(A34) $$P_{\mathrm{PD},t}=P\left(\left(C_{x,t},C_{y,t}\right)\in R_{\mathrm{PD}}\right)$$

where

(A35) $$R_{\mathrm{PD}}=\left\{\left(0,\;33\right),\left(-1,\;33\right),\left(-1,\;24.5\right),\left(0,\;24.5\right)\right\}$$

(A36) $$P_{D1,t}=P\left(\left(C_{x,t},C_{y,t}\right)\in R_{D1}\right)$$

where

(A37) $$R_{D1}=\left\{\left(0,\;40\right),\left(38,\;12\right),\left(32,\;-6.1\right),\left(4,\;16\right),\left(0,\;1.5\right)\right\}$$

(A38) $$P_{D2,t}=P\left(\left(C_{x,t},C_{y,t}\right)\in R_{D2}\right)$$

where

(A39) $$R_{D2}=\left\{\left(4,\;16\right),\left(32,\;-6.1\right),\left(24.3,\;-30\right),\left(0,\;-3\right),\left(0,\;1.5\right)\right\}$$

(A40) $$P_{S,t}=P\left(\left(C_{x,t},C_{y,t}\right)\in R_{S}\right)$$

where

(A41) $$\begin{array}{c} R_{S}=\left\{\left(0,\;1.5\right),\left(-35,\;-3.1\right),\left(-38,\;12.4\right),\left(0,\;40\right),\right.\left(0,\;33\right),\\ \left.\left(-1,\;33\right),\left(-1,\;24.5\right),\left(0,\;24.5\right)\right\} \end{array}$$

(A42) $$P_{T3-H,t}=P\left(\left(C_{x,t},C_{y,t}\right)\in R_{T3-H}\right)$$

where

(A43) $$\begin{array}{c} R_{T3-H}=\left\{\left(0,\;-3\right),\left(24.3,\;-30\right),\left(23.5,\;-32.4\right)\right.,\\ \left.\left(2.5,\;-32.4\right),\left(-3.5,\;-3\right)\right\} \end{array}$$

(A44) $$P_{C,t}=P\left(\left(C_{x,t},C_{y,t}\right)\in R_{C}\right)$$

where

(A45) $$R_{C}=\left\{\left(-3.5,\;-3\right),\left(2.5,\;-32.4\right),\left(-21.5,\;-32.4\right),\left(-11,\;-8\right)\right\}$$

(A46) $$P_{O,t}=P\left(\left(C_{x,t},C_{y,t}\right)\in R_{S}\right)$$

where

(A47) $$\begin{array}{c} R_{O}=\left\{\left(-3.5,\;-3\right),\left(-11,\;-8\right),\left(-21.5,\;-32.4\right),\left(-23.5,\;-32.4\right),\right.\\ \left(-35,\;3.1\right),\left.\left(0,\;1.5\right),\left(0,\;-3\right)\right\} \end{array}$$
